# Stimulation uncertainty reveals distinct mechanisms of sensation seeking and impulsivity

**DOI:** 10.1038/s44271-026-00525-5

**Published:** 2026-10-23

**Authors:** Ern Wong, Tobias U. Hauser, Aswath Chandrasekaran, Pietro Pietrini, Charley M. Wu

**Affiliations:** 1 MoMiLab, IMT School for Advanced Studies Lucca, Lucca, Italy; 2 Human and Machine Cognition Lab, Technische Universität Darmstadt, Darmstadt, Germany; 3 Department of Psychiatry and Psychotherapy, Faculty of Medicine, University of Tübingen, Tübingen, Germany; 4 German Centre for Mental Health (DZPG), Tübingen, Germany; 5 Max Planck UCL Centre for Computational Psychiatry and Ageing Research, University College London, London, UK; 6 Center for Cognitive Science, Technische Universität Darmstadt, Darmstadt, Germany; 7 Hessian AI, Darmstadt, Germany

**Keywords:** Human behaviour, Decision making, Human behaviour

## Abstract

Sensation-seeking is often described as a tendency to pursue novel and stimulating experiences, yet such options are often uncertain as well. This creates a central ambiguity where choices that appear to reflect attraction to stimulation may instead reflect tolerance of uncertain outcomes, or an interaction between the two. Across two experiments, we tested how expected stimulation and stimulation uncertainty shape reward-guided choice. A reanalysis of a previous operant sensation-seeking dataset suggests that stimulation uncertainty explained behaviour better than expected stimulation. Subsequently, we conducted a preregistered online experiment to investigate the joint effects of expected stimulation and stimulation uncertainty and to compare their respective associations with sensation-seeking and impulsivity. Our results indicate that higher sensation-seeking was associated with broader stochastic sampling and lower reward performance, but this was not reducible to random choice or uncertainty tolerance alone. Instead, uncertainty weighting depended on the value assigned to expected stimulation. This profile differed from impulsivity, suggesting that the two traits shape choice through distinct computational routes. By separating expected stimulation from uncertainty, these findings refine accounts of exploratory choice by showing that sensation-seeking shapes not only how much people sample uncertain options, but how stimulation value and uncertainty are jointly weighted during choice.

## Abstract (web summary)

Classical accounts of sensation seeking emphasise attraction to stimulating outcomes. This study examines the complementary role of uncertainty, showing that sensation seekers seek uncertainty when stimulation value is low but avoid it when value is high, a profile distinct from impulsivity.

## Introduction

Sensation-seeking is a personality trait characterised by the pursuit of “varied, novel, complex, and intense sensations and experiences,” even when these experiences carry potential costs^1^. It is associated with a broad range of exploratory and risky behaviours^2,3^, including alcohol and substance use^4,5^, antisocial behaviour^6,7^, and other maladaptive outcomes with substantial individual and societal costs^8,9^. Yet sensation-seeking is not uniformly harmful. Higher sensation-seeking has also been linked to better psychological well-being^10,11^ and to forms of positive risk-taking, such as social exploration and pursuing challenging goals^12–16^. This duality suggests that sensation-seeking is not simply a general liability for risky behaviour, nor is it inherently beneficial. Its consequences may depend on the structure of the decision environment— whether novel or stimulating options provide useful information or long-term value, or instead compete with more immediately rewarding goals.

A prominent mechanistic explanation is that sensation-seeking reflects heightened valuation of stimulation. On this *expected-stimulation account*, high sensation-seekers assign greater subjective utility to actions that produce stimulating experiences^17–19^. This view is consistent with neurobiological accounts linking sensation-seeking to dopaminergic systems involved in incentive salience and reward learning^17^, as well as evidence that high sensation-seekers show distinct dopaminergic profiles, including elevated dopamine in the caudate nucleus and attenuated dopamine turnover rates^20^. However, these findings leave open other computational mechanisms through which sensation-seeking may shape choice. In particular, they do not establish whether high sensation-seekers are attracted to stimulation itself or whether they are more willing to choose options whose stimulation outcomes are *uncertain*.

This distinction is crucial because options that promise stimulation are often uncertain as well. Novel, complex, or ambiguous stimuli have long been theorised to carry arousal potential and to motivate exploratory behaviour^21^. In reinforcement-learning accounts, uncertain options can attract choice because sampling them may provide useful information for future decisions, a process often described as directed exploration^22,23^. Related accounts of information seeking propose that information can carry subjective value even when it is not immediately reward-maximising^24,25^. While sensation-seeking may not be exactly identical to directed exploration, the two constructs overlap in one important respect: novel or stimulating options are generally unusual, and often require a degree of exploration to obtain^8^. High sensation-seekers may therefore either approach uncertain options because uncertainty carries potential informational value (which may or may not be immediately reward-maximising), or because the possibility of stimulation is motivating in itself. These alternatives are difficult to distinguish from choice behaviour alone. Choices toward stimulating options may reflect attraction to expected stimulation, tolerance of uncertain stimulation, or a context-dependent combination of the two.

This context-dependent view connects sensation-seeking to broader accounts of its functional and dysfunctional expressions. The same tendency to approach novel or stimulating experiences may support exploration and opportunity-seeking in some environments, but contribute to costly risk-taking in others^8^. One possible reason is that uncertainty itself can change meaning across contexts. When uncertain options provide information, opportunity, or future value, sampling them may resemble curiosity-driven exploration^25,26^. When uncertainty is non-instrumental, however, the same sampling tendency may draw behaviour away from more reliable goals. Understanding sensation-seeking, therefore, requires identifying not only whether high sensation-seekers value stimulation, but also whether and when they tolerate uncertainty about stimulation.

Here, we address this ambiguity by separating expected stimulation from stimulation uncertainty. Expected stimulation refers to the learned probability that an option will produce stimulation. Stimulation uncertainty refers to uncertainty about whether stimulation will occur, which is highest when stimulation is maximally unpredictable and lowest when stimulation is either very likely or very unlikely. These quantities are often correlated in real-world settings and in existing task designs: options that are novel, exciting, or poorly known may also be those with uncertain outcomes. As a result, apparent stimulation-seeking can be confounded with uncertainty tolerance. A design that separates expected stimulation from stimulation uncertainty is therefore needed to test whether sensation-seeking related choice is driven by expected stimulation, uncertainty, or their interaction.

A second challenge is that sensation-seeking is often discussed alongside impulsivity. Although both traits are associated with risk-taking, they appear to capture different routes to risky and exploratory behaviour^3,27–31^. Sensation-seeking and impulsivity are only modestly correlated and show different associations with real-world outcomes^28,32^. For example, sensation-seeking is more closely linked to initial experimentation with alcohol and drugs, whereas impulsivity is more strongly associated with escalation, compulsive use, and difficulty sustaining goal-directed behaviour^33–35^. This makes impulsivity a critical comparison trait. If sensitivity to expected stimulation or stimulation uncertainty reflects generic risk propensity, poor behavioural control, or noisy choice, similar associations should emerge for impulsivity as well. By contrast, dissociable sensation-seeking and impulsivity profiles would suggest that these traits influence risky or exploratory choice through distinct decision mechanisms.

In the present study, we tested whether sensation-seeking-related choice is better characterised by expected stimulation, uncertainty about stimulation, or their joint contribution. We used a two-part approach. In Experiment 1, we reanalysed a previous operant sensation-seeking dataset in which participants chose between options associated with reward and probabilistic sensory stimulation^17^. This allowed us to ask whether previously reported sensation-seeking effects were better captured by learned expected stimulation or by uncertainty about stimulation. However, because expected stimulation and stimulation uncertainty were correlated in that task, Experiment 1 could not decisively separate these mechanisms. Experiment 2, therefore, used a preregistered web-based task designed to reduce the collinearity between expected stimulation and stimulation uncertainty^36^. Participants first learned stable reward contingencies and then made choices during a stimulation phase in which stimulation probability varied independently of reward. This design allowed us to estimate the unique and joint contributions of expected stimulation and stimulation uncertainty to choice. Across both experiments, we additionally compared sensation-seeking with impulsivity to test whether stimulation-related uncertainty tolerance reflects a sensation-seeking-specific profile rather than a broader risk-related tendency.

## Methods

### Participants

#### Experiment 1

We reanalysed two previously collected and reported datasets by Norbury et al.^17^. At the time of collection, all participants provided written informed consent, and the study was approved by the University College London Ethics Committee. Below, we provide a brief overview of the participant samples relevant to the present analyses; further recruitment and eligibility details are available in the original publication.

Dataset 1 recruited 45 healthy adults (28 female, 17 male; *M*_age_ = 24.3 ± 3.55 years; age range = [20 − 36] years). Sensation-seeking scores were unavailable for 6 participants, leaving *n* = 39 participants with complete trait data. Dataset 2 comprised a pharmacological study that recruited 30 healthy male participants (*M*_age_ = 22.3 ± 2.74 years; age range = [19 − 29] years). Two participants were excluded from the original study—one for below-criterion-level performance and another for a final-session dropout, yielding *n* = 28. Self-reported gender information was obtained from the original study records, using the categories reported above. Socioeconomic status was not directly measured; however, years of education were available for Dataset 1 (*M*_education_ = 16.1 ± 3.1 years), but not for Dataset 2. Information on communities of descent and race/ethnicity was not collected. Behavioural data from the two datasets were pooled for the present reanalysis, yielding *n* = 73 participants for analyses that did not require sensation-seeking scores. Analyses involving sensation seeking were restricted to the *n* = 67 participants with available trait data. Sensation seeking was assessed using the revised Sensation-Seeking Scale (SSS-V)^35,38^.

#### Experiment 2

A total of *n* = 619 participants were recruited through Prolific. The study was approved by the Ethics in Psychological Research Commission of the University of Tübingen (Wu_2021/0124/213). All participants provided informed consent prior to participation. All procedures were conducted in accordance with the Declaration of Helsinki and relevant institutional guidelines. Eligibility criteria required participants to be aged 18 years or older, reside in either the United Kingdom or the United States, and have a Prolific approval rate of at least 95%. Participants were excluded based on instructional manipulation checks and a performance criterion. Eighty-four participants were excluded for failing at least one of the two instructional manipulation checks embedded in the questionnaires. An additional 8 participants were excluded for performing below chance in Phase 1, defined as achieving less than 50% of the Phase-1 maximum score. This performance criterion deviated from the preregistered exclusion criterion and was adopted to provide a more targeted measure of reward-contingency learning; full details and rationale are provided in Supplementary Table S1.

The final sample, therefore, consisted of *n* = 527 participants (264 female, 263 male; *M*_age_ = 39.8 ± 13.0 years; age range = [18−80] years). Gender was self-reported by participants in the demographic questionnaire using the categories above. Socioeconomic status, communities of descent, and race/ethnicity were not collected. Participants received a base payment of €5 and could earn an additional performance-based bonus of up to €5, determined by their performance in one randomly selected round.

### Preregistration

Experiment 1 was a reanalysis of published data^17^ and was not preregistered. For Experiment 2, the research question, hypotheses, exclusion criteria, and planned analyses were preregistered on 14 June 2025 on the Open Science Framework (OSF; https://doi.org/10.17605/OSF.IO/H4P3T)^36^. The preregistered hypotheses and analyses, together with any deviations from the preregistration, are summarised in Supplementary Table S1.

### Study Design

#### Experiment 1

Participants completed a two-phase, two-armed bandit task designed to measure the value assigned to stimulation. Here, sensory stimulation was a mild, non-aversive electric stimulation delivered to the hand. The task comprised a reward-learning phase followed by a test phase with stimulation. During reward learning, participants learned the point values associated with eight bandits. Two bandits were assigned to each of four fixed payoffs (25, 50, 75, or 100 points). On every trial, two bandits were presented side by side, and one choice was required; this pairing scheme produced 10 unique pair types (combinatorial pairs of the four payoffs, with two exemplars per payoff). The phase continued for at least 80 trials and progressed until a prespecified performance criterion was met, ensuring adequate learning of the reward structure prior to the introduction of stimulation.

In the subsequent test phase, half of the bandits were designated stimulation-paired (probability of stimulation = 0.75) and half non-stimulation (probability = 0). Each test trial paired one stimulation-paired bandit with one non-stimulation bandit, yielding three payoff scenarios in equal proportion: the stimulation-paired bandit offered a higher, lower, or equal point payoff relative to its partner. Participants completed 100 test trials (10 per trial type), allowing us to estimate preferences for stimulation-paired options conditional on relative monetary value. Prior to any task exposure, participants provided liking ratings for each bandit on a continuous visual analogue scale. Identical ratings were collected again after reward learning and after the test phase. The change from post-learning to post-test indexed overall stimulation liking at the end of the experiment.

#### Experiment 2

**Fig. 1:**
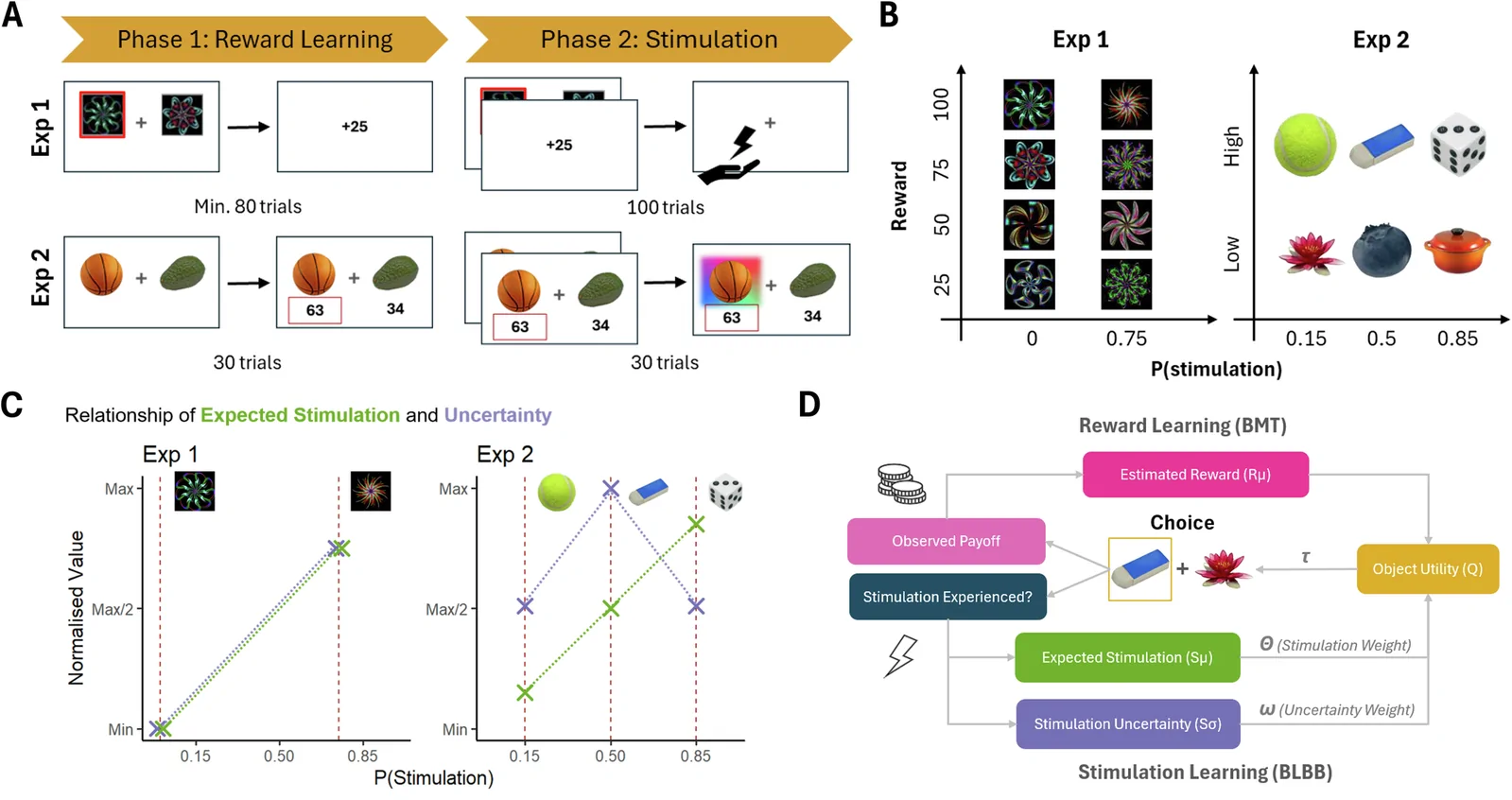
Task description and model overview. **A** Task overview. In both experiments, participants completed a reward-learning phase followed by a stimulation phase. In *Experiment 1*, participants first learned the reward payoffs of eight bandits until they reached a performance criterion. In the test phase, four bandits were associated with stimulation and four with no stimulation; each trial paired a stimulation bandit with a non-stimulation bandit. In *Experiment 2*, participants completed four rounds of the same two-phase structure (60 trials per round) with six objects per round defined by crossing reward level with stimulation probability. Rewards were learned with full feedback, then the same reward contingencies were tested under stimulation, with all object pairs presented repeatedly in random order. **B** Different stimulus sets were used for Experiments 1 and 2. *Experiment 1* used eight fractals parameterised by payoff (25/50/75/100) and stimulation assignment (*p* = 0 or 0.75); *Experiment 2* used six Bank of Standardised Stimuli (BOSS)^39,40^ images parameterised by reward level (low/high) and stimulation probability (0.15/0.50/0.85). **C** Sampling scheme. In *Experiment 1*, the limited probability set caused *expected stimulation* (green) and *uncertainty* (purple) to be correlated. *Experiment 2* removed this confound by sampling a wider range of probabilities, thereby reducing the collinearity between expected stimulation probability and stimulation uncertainty. **D** Computational model schematic: object utility (*U*) depends on estimated reward (*Rμ*) and contributions from stimulation mean (*Sμ*) weighted by *θ*, and uncertainty (*Sσ*) weighted by *ω*, given observed payoffs and whether stimulation occurred. Object utilities are then passed through a softmax controlled by temperature *τ*, which governs the stochasticity of the choice. These quantities are updated using the learning rules described in the main text. BMT Bayesian Mean Tracker, BLBB Bayesian Learner Beta-Binomial.

Experiment 2 (Fig. 1B) was a new, preregistered experiment^36^ designed to reduce the collinearity between expected stimulation and stimulation uncertainty that constrained their interpretation in Experiment 1 (Fig. 1C). This allowed us to examine their unique and joint contributions to choice and their relationships with sensation seeking and impulsivity.

Participants completed four rounds of a web-based adaptation of the operant sensation task^17,18^. Similarly, each round consists of Phase 1 (reward learning) and Phase 2 (test with stimulation), totalling 60 trials per round (30 trials per phase). In this paradigm, participants could self-administer visual stimulation (a coloured spinning animation; 1.4s) while engaging in a two-armed bandit task. On every trial, participants had up to 6 seconds to respond; non-responses were recorded as missed trials. Before proceeding with the main task, participants provided informed consent, read on-screen instructions, and completed four comprehension questions; all questions had to be answered correctly to proceed.

At the start of each round, we sampled six object images from the Bank of Standardized Stimuli (BOSS)^37,40^ and assigned two orthogonal parameters to each object. First, an unscaled reward level was sampled from either a high-reward distribution (*N*~(10, 2.5)) or a low (*N*~(−10, 2.5)) distribution, and a stimulation probability (0.15, 0.50, or 0.85). Assignments were fixed within a round and balanced so that across the six objects, the reward factor and stimulation-probability factor were independent by design, enabling separate identification of effects due to expected stimulation probability and stimulation uncertainty. Phase 1 (reward learning) was explicitly structured to minimise epistemic sampling for reward: on each of 30 trials, participants chose between two of the six objects and then received full feedback—the realised reward for the chosen and the unchosen object—expediting belief formation about object values. Phase 2 (test with stimulation, here termed “glitch") introduced stimulation while keeping the learned reward contingencies unchanged. In each of 30 trials, the visual stimulus was delivered to the chosen object with its assigned probability (0.15, 0.50, or 0.85). Participants were reminded at the transition that stimulation does not influence rewards, which remained governed solely by the Phase-1 reward structure. Within each round, we pre-generated all 15 unique object pairs (6 choose 2) and presented each pair exactly four times, yielding 60 trials. The order of pair presentation and left/right positions was independently randomised for each participant across trials. This scheme ensured that each object appeared in a comparable number of pairings, preserved an identical pairing structure across participants (with only trial order randomised), and controlled for spatial biases.

We applied a random scaling of rewards across rounds (i.e., different minimum and maximum rewards) to prevent participants from immediately recognising when they had chosen the optimal option. For each round, we sampled a value from a uniform distribution *U* ~ (1, 5), which scaled the rewards after shifting them by 10. We also truncated rewards to ensure they were always non-negative. In order to convey intuitions about the random scaling of rewards, payoffs were presented using a different fictional currency in each round, such that the absolute value was unknown, but higher payoffs were always better. Rewards were transformed according to payoff = (rawScore + 10)*s*, where *s* ~ *U*(1, 5).

Following each round, participants provided subjective ratings of stimulation valence (unpleasant-pleasant) and arousal (sleepy-wakeful) on Likert scales [0–100]. The task was delivered online with standard display instructions (full-screen mode encouraged); responses were collected via keyboard, and reaction times were recorded on each trial.

#### Psychiatric questionnaires

After completing the task, participants completed several questionnaires assessing psychiatric dimensions and general cognitive ability. These include the Sensation Seeking Scale V^35,38^ (SSS-V), Barratt Impulsiveness Scale^41^ (BIS-21), Depression Anxiety and Stress Scale 21^42^ (DASS-21), Obsessional Compulsive Inventory - Revised^43^ (OCI-R), Alcohol Use Disorders Identification Test^44^ (AUDIT), Drug Abuse Screening Test^45^ (DAST-10), and the International Cognitive Ability Resource^46^ (ICAR-16). To ensure data quality, instructional manipulation checks^47^ (IMCs) were included as attentional checks. Participants failing any one of these IMCs were excluded from the analysis.

### Statistics and Reproducibility

Analyses were conducted in R (version 4.4.1) using RStudio. Hierarchical generative modelling was carried out with rstan^48^ (version 2.32.6). Mixed-effects regressions were performed using lme4^49^ (version 1.1-35.5). Regression model comparisons were conducted with the performance package^50^ (version 0.15.0). Johnson–Neyman interaction analyses were conducted using emmeans^51^ (version 1.10.4). Joint path modelling was performed using lavaan^52^ (version 0.6.21). All statistical tests were two-sided. No adjustments for multiple comparisons were applied. Sample sizes and participant exclusions are reported in the Participants section. Individual participants were independent observational units, with repeated trials and rounds within each participant treated as repeated measures.

Assumptions were assessed separately for each analysis. Pearson correlations were examined for linearity, residual normality, homoscedasticity, leverage, and influential observations using scatterplots and regression diagnostics; no substantial violations were identified. Regression models were assessed for relevant assumptions, including linearity, homoscedasticity, residual normality, multicollinearity, and influential observations where applicable. Mixed-effects models were additionally assessed for convergence and singularity, and binomial logistic mixed-effects models for overdispersion and systematic patterns in simulated residuals using the DHARMa package^53^, with no substantial violations identified.

#### Computational modeling

We model trialwise choices as the integration of three components: (i) expected point payoff, (ii) Beliefs about stimulation (its expected mean probability and uncertainty), and (iii) a value-free tendency to repeat either the previous button pressed (motor stickiness) or the previous bandit selected (bandit stickiness). Beliefs about expected payoff and stimulation are updated by learning rules elaborated below.

**Reward Learning (Bayesian Mean Tracker)**. In Experiment 1, we retained the assumption of the original authors that rewards were fully learned and treated as fixed following the training phase. Accordingly, object payoff values *Rμ*_*j*_ were modelled as known constants (i.e. no trial-wise updating).

In contrast, the reward payoffs in Experiment 2 were Gaussian but stationary. To capture participants’ evolving expectations in this environment, we employed a Bayesian Mean Tracker (BMT)^54–56^, a type of Kalman filter specifically designed for stationary reward settings. Briefly, for each bandit *j* we track a normal posterior:

1 $$P(R{\mu }_{j,t}| {{{{\mathcal{D}}}}}_{t})={{{\mathcal{N}}}}(R{\mu }_{j,t},R{\sigma }_{j,t})$$

The posterior mean *Rμ*_*j*,*t*_ and variance *Rσ*_*j*,*t*_ are updated iteratively based on observed rewards *y*_*t*_. In our experiment setup, the posterior is updated for both chosen and unchosen bandits due to complete feedback.

2 $$R{\mu }_{j,t}=R{\mu }_{j,t-1}+{G}_{j,t}\left[{y}_{t}-R{\mu }_{j,t-1}\right]$$

3 $$R{\sigma }_{j,t}=\left[1-{G}_{j,t}\right]R{\sigma }_{j,t-1}$$

Here, the Kalman Gain *G*_*j*,*t*_ modulates the size of each update and is calculated as:

4 $$\begin{array}{rc}{G}_{j,t}&=\frac{R{\sigma }_{j,t-1}}{R{\sigma }_{j,t-1}+{\zeta }_{\epsilon }^{2}}\end{array}$$

The parameter ${\zeta }_{\epsilon }^{2}$ represents the error variance, controlling the model’s sensitivity to new information. Lower values of ${\zeta }_{\epsilon }^{2}$ result in larger updates and faster uncertainty reduction. Thus, the BMT offers a flexible, uncertainty-aware framework for modelling learning in environments with stationary reward distributions.

**Stimulation Learning**. We implemented a Bayesian model that assumes stimulation probabilities for each object *j* are represented as beta distributions. This approach is most appropriate for learning probabilities since the distribution is between 0 and 1, and the variance estimates uncertainty. Similar models have previously been used to model value-based learning^57^ and aversive learning tasks^58,59^.

5 $${P}_{j,t}(stim) \sim Beta({\alpha }_{j,t},{\beta }_{j,t})$$

Intuitively, the model describes how the evidence for stimulation depends on the number of prior stimulations. These counts can be represented for each bandit by the parameter *α*_*j*_, which is incremented depending on whether a stimulation is observed (*S*_*j*,*t*_ = 1). Similarly, the counts of no stimulation (*S*_*j*,*t*_ = 0) are tracked by a complementary parameter *β*_*j*_. Both *α* and *β* are initialised as 1. For simplicity, we assume optimal updating, where *α* and *β* are updated by a value of 1 after observing the respective outcome:

6 $${\alpha }_{j,t+1}={\alpha }_{j,t}+{S}_{j,t}$$

7 $${\beta }_{j,t+1}={\beta }_{j,t}+(1-{S}_{j,t})$$

The expected probability of stimulation *Sμ* for bandit *j* at trial *t* is:

8 $$S{\mu }_{j,t}=\frac{{\alpha }_{j,t}}{{\alpha }_{j,t}+{\beta }_{j,t}}$$

and the associated uncertainty *Sσ* is:

9 $$S{\sigma }_{j,t}=\sqrt{\frac{{\alpha }_{j,t}{\beta }_{j,t}}{{({\alpha }_{j,t}+{\beta }_{j,t})}^{2}({\alpha }_{j,t}+{\beta }_{j,t}+1)}}$$

##### Value-free stickiness

We compare two value-free perseveration effects, added *outside* the temperature scaling: motor (repeat the previous *button*, weighted by *ρ*_*m*_) and stimulus (repeat the previous *object identity*, weighted by *ρ*_*s*_).

Given *a*_*t*−1_ ∈ {L, R} as the previous action and *o*_*t*−1_ the previously chosen object (so *o*_*t*−1_ = *o*_*t*−1_(*a*_*t*−1_)),

10 $${\rho }_{t}^{\,{{\rm{m}}}\,}(a)={\rho }_{m}\,{\mathbb{I}}[\,a={a}_{t-1}\,],$$

11 $${\rho }_{t}^{\,{{\rm{s}}}\,}(a)={\rho }_{s}\,{\mathbb{I}}[\,{o}_{t}(a)={o}_{t-1}\,],$$

with ${\rho }_{1}^{\cdot }(a)=0$ on the first trial. Intuitively, this assigns a bonus to either the chosen button or the bandit from the preceding trial, with the magnitude and direction of the effect governed by *ρ*_*m*_ or *ρ*_*s*_, respectively.

**Decision Rule**. Q-values for each bandit *j* were calculated as a linear sum of their estimated underlying point payoff *Rμ*_*j*_, the expected mean probability of stimulation, *Sμ*_*j*_, scaled by a free parameter *θ* weight, and the associated uncertainty *Sσ*_*j*_, scaled by a free parameter *ω* weight.

12 $${Q}_{j,t}=R{\mu }_{j,t}+\theta S{\mu }_{j,t}+\omega S{\sigma }_{j,t}$$

Choice probabilities were determined by a softmax function incorporating the Q-value of each bandit, a temperature parameter *τ* governing choice stochasticity and a stickiness bonus *ρ*_*j*,*t*_ denoting the corresponding motor- or stimulus-stickiness bonus for bandit *j* on trial *t*, depending on the model specification

13 $$P({{\mbox{choice}}}_{t}=A)=\frac{\exp \left(\frac{{Q}_{A,t}}{\tau }+{\rho }_{A,t}\right)}{\exp \left(\frac{{Q}_{A,t}}{\tau }+{\rho }_{A,t}\right)+\exp \left(\frac{{Q}_{B,t}}{\tau }+{\rho }_{B,t}\right)}.$$

For brevity, the full model (*R*−*θωρ*) is described as above. We also compared reduced versions of the model via model ablation. In Experiment 1, we compared single-process models against each other, i.e., *R*−*θ* and *R* − *ω* (See Supplementary Information for more details on reduced models).

#### Mixed Effect Regressions

We quantified how reward and stimulation signals shaped choice using trial-level mixed-effects logistic regression. For each trial, we constructed difference predictors as the right minus left model estimates (differences are denoted by Δ): expected reward (Δ*Rμ*), expected stimulation (Δ*Sμ*), and stimulation uncertainty (Δ*Sσ*). Trials with no response were excluded. All continuous predictors and trait scores were z-scored. Our preregistered primary model predicted the probability of choosing the right option with a logit link, including fixed effects of Δ*Rμ*, Δ*Sμ*, and Δ*Sσ*, and a random intercept and random Δ*Rμ* slope by participant, which allowed value sensitivity to vary across individuals:

P(choice_*i,t*_ = Right)  ~ Δ*Rμ*_*i,t*_ + Δ*Sμ*_*i,t*_ + Δ*Sσ*_*i,t*_ + Sticky_*i,t*_ + (1 + Δ*Rμ*_*i,t*_ ∣ Subject_i_)

We also evaluated the two alternative versions of stickiness as mentioned above and compared them using AIC/BIC. The final trait-moderation analyses retained the preregistered value and stimulation predictors, with motor stickiness (Table S3).

To further test whether traits modulate these computational signals, we added cross-level interactions with sensation-seeking and impulsivity, entered as z-scores. All lower-order terms were included.

Models were fit in lme4::glmer (binomial family, logit link); convergence and singularity were checked. We report the *β* coefficients with 95% CIs, and compared random-effects structures and fixed-effect subsets using AIC and likelihood-ratio tests where appropriate. Model diagnostics included overdispersion checks and DHARMa residual simulations. For statistically significant higher-order interactions, we probed simple slopes (estimated marginal trends) at representative moderator levels and, where relevant, Johnson-Neyman regions restricted to the observed range of the moderator.

### Generative model fitting and comparison

All models were estimated hierarchically using custom-written Stan code. Specifically, we used Hamiltonian MCMC with a No-U-Turn sampler to estimate group-level location and scale parameters for all model parameters across participants. Group-level location parameters for expected-stimulation weighting (*θ*), stimulation-uncertainty weighting (*ω*), stickiness (*ρ*), and log inverse temperature were assigned weakly informative ${{{\mathcal{N}}}}(0,1)$ priors. Group-level scale parameters were constrained to be positive and assigned half-normal ${{{{\mathcal{N}}}}}^{+}(0,1)$ priors, with participant-level parameters estimated using a non-centred parameterisation with standard-normal latent deviations. These priors were chosen to provide mild regularisation while remaining broad relative to the plausible range of the parameters and were not intended to encode substantive hypotheses. The choice model was parameterised using inverse temperature; for interpretation and reporting, posterior draws were transformed to the conventional temperature parameter (*τ*) by taking their reciprocal, such that larger *τ* values indicate greater choice stochasticity. Posterior distributions of group-level parameters were summarised using the posterior mean and 95% credible interval (See Table S4).

Chain convergence was assessed using the $\widehat{R}$ statistic, where $\widehat{R}\le 1.01$ was considered acceptable, together with effective sample size diagnostics and checks for divergent transitions. The model was estimated over 4 chains of 4000 iterations, with the first 1000 iterations per chain used for warm-up and a target acceptance probability of 0.99. The point payoff for all bandits was scaled to the range [0, 1] before fitting.

Model comparison was conducted using the loo package in R, leveraging a version of the loo estimate optimised through Pareto-smoothed importance sampling (PSIS) methodology^60^. The loo approach assesses the out-of-sample predictive accuracy of the model by evaluating how well the model predicts the outcome for each data point using the entire dataset, excluding that point.

Posterior predictive checks were conducted for the winning model by drawing posterior samples of participant-level parameters and simulating choices from the fitted likelihood. Simulated choices were summarised in the same way as observed choices: trial-level choice curves were computed after binning trials by Δ*Rμ*, Δ*Sμ*, and Δ*Sσ*, and behavioural summaries were computed across sensation-seeking and impulsivity quartiles. For each summary, we report the posterior predictive mean and 95% predictive interval (See Fig. S9).

### Linking model parameters to traits

We related individual differences in the hierarchical model to questionnaire traits in two steps. First, we quantified partial correlations between trait scores and subject-level parameters from the hierarchical Bayesian choice model, *θ*, *ω*, *τ*, and *ρ*. For interpretability, we analysed $\log (\tau )$. All variables were z-scored prior to analysis. Partial correlations were computed using Pearson’s r, adjusting for age, IQ, gender, and the other trait (i.e., sensation-seeking partials controlled for impulsivity and vice versa).

Second, to test whether traits reflect a joint contribution of value and uncertainty mechanisms, we fit subject-level linear regressions with all lower-order terms and the three-way interaction between $\omega ,\theta ,and\log (\tau )$, while adjusting for covariates (age, IQ, gender) and the other trait. We did not include *ρ* (motor stickiness) in the interaction space because our a priori question targeted the synergy between expected-stimulation weighting (*θ*) and uncertainty (*ω*); adding *ρ* interactions would inflate the model without clear theoretical gain and reduce power. As a robustness check, adding *ρ* as an additional covariate did not substantially change our results.

The models were:

Sensation seeking ~ $\omega \times \theta \times \log (\tau )$ + impulsivity + age + IQ + gender

Impulsivity ~ $\omega \times \theta \times \log (\tau )$ + sensation seeking + age + IQ + gender

Model diagnostics included residual inspection and variance-inflation factors (all *VIFs* < 5). When higher-order interactions were significant, we probed simple slopes using standard linear-model contrasts and Johnson-Neyman intervals, expressly restricting inference to the observed moderator range.

To directly compare parameter–trait associations for sensation-seeking and impulsivity, we fitted a joint path model in which both traits were simultaneously regressed on the same computational parameters, interaction terms, and covariates. The residual covariance between sensation-seeking and impulsivity was freely estimated. Because this model was saturated, global fit indices were not interpreted; inference focused on predefined contrasts comparing corresponding sensation-seeking and impulsivity paths.

## Results

We report our results in two parts. Experiment 1 reanalyses prior data^17^ to test whether previously reported sensation-seeking effects may be better explained by expected stimulation or by stimulation uncertainty. Because these quantities were correlated in the original design, Experiment 2 used a preregistered experiment^36^ with an orthogonalised design, allowing us to estimate the unique and joint contributions of expected stimulation and stimulation uncertainty on choice, and to directly compare their associations with sensation-seeking and impulsivity.

### Experiment 1: Prior sensation-seeking effects are better captured by stimulation uncertainty

We reanalysed a previously collected dataset^17^ to investigate whether sensation-seeking is more closely related to responses to stimulation *itself* (expected probability, *Sμ*) or to stimulation *uncertainty* (variance around that probability, *Sσ*). This was an exploratory reanalysis and was not preregistered. Here, participants completed an operant two-armed bandit task in which they chose between abstract stimuli associated with different reward values and probabilistic delivery of non-painful electrical stimulation. Unlike the original analysis, which represented stimulation contingencies as fully known stimulus features, we fit hierarchical generative Bayesian learning models (Bayesian Learner Beta Binomial, BLBB; see Methods for details) that updated beliefs about stimulation probability over trials.

**Fig. 2:**
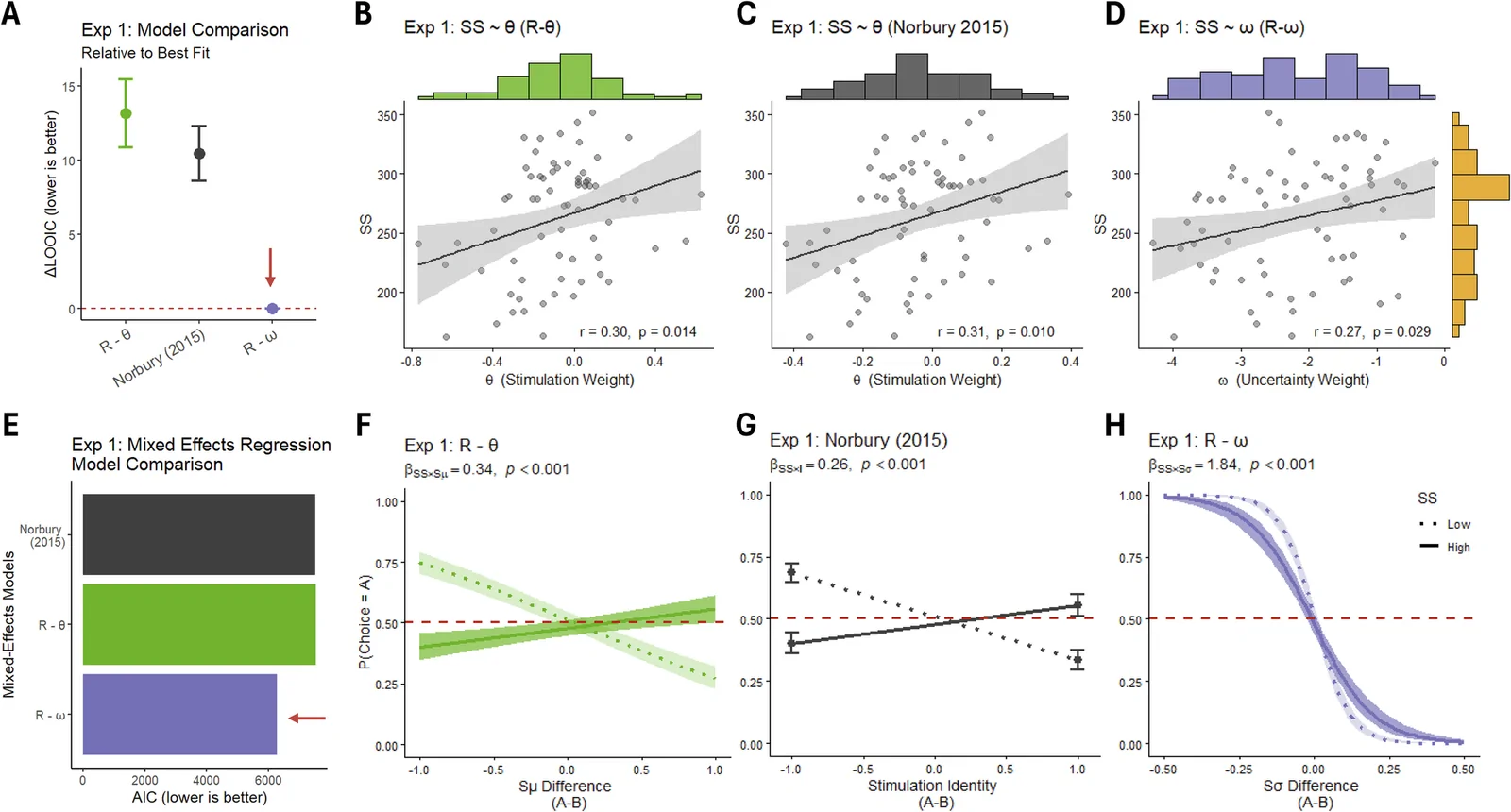
Experiment 1: re-analysis and model comparison. We re-analysed Norbury et al. (2015)^17^ to test whether SS is better captured by an expected stimulation or a stimulation uncertainty account. Their respective contributions are captured using weights on the modelled decision variables: *θ* for the learned expected stimulation probability and *ω* for stimulation uncertainty. A Bayesian Learner Beta-Binomial provided trial-wise estimates of stimulation probability and uncertainty; choices were fit with single-process utilities---expected stimulation (*R* − *θ*) and uncertainty (*R* − *ω*)---and compared with the original Norbury model. **A** Out-of-sample predictive fit (LOO, relative to best) favoured *R* − *ω* (*n* = 73). **B**–**H** Analyses involving SS scores included *n* = 67 participants. **B** Replicating the original expected stimulation effect with our learner: SS correlated with *θ* (two-sided Pearson correlation: *t*(65) = 2.52, *r* = . 30, 95% *CI* [0.06, 0.50], *p* = . 014), though this was not the best-fitting model. **C** Replication using the Norbury (2015) model with hierarchical Bayesian fitting (*t*(65) = 2.67, *r* = 0.31, 95% *CI* [0.08, 0.52], *p* = 0.010). **D** From the best-fitting model, SS also correlated with the uncertainty weight *ω* (*t*(65) = 2.23, *r* = 0.27, 95% *CI* [0.03, 0.48], *p* = 0.029); because *ω* was negative on average, higher SS is associated with greater tolerance for uncertainty. **E** Mixed-effects regression comparison (AIC): models including stimulation uncertainty provided the best fit. We visualise the interaction of either stimulation mean (*Sμ*) or uncertainty (*Sσ*) with SS at ±2*SD*. **F** *Sμ* × SS interaction under the *R*−*θ* model (*β*_*Sμ* ×* SS*_ = 0.34,  95% *CI* [0.25, 0.42],  *z* = 7.74,  *p* = 9.81 × 10^−15^). **G** *Sμ* × SS interaction under the Norbury (2015) model (${\beta }_{{S}_{Identity}\times SS}=0.26,\,95 \% \,CI\,[0.2,0.3],\,z=9.31,\,p < 2.00\times 1{0}^{-16}$). **H** *Sσ* × SS interaction under the *R*−*ω* model (*β*_*Sσ*__×__*SS*_ = 1.84,  95% *CI* [1.0, 2.7], *z* = 4.26,  *p* = 2.06 × 10^−5^). Note. Stimulation mean and uncertainty were collinear in this design, so models were evaluated separately; a joint model is tested in Experiment 2, where these factors were orthogonalised. All statistical tests were two-sided. No adjustments for multiple comparisons were applied. Error bars in (**A**) represent ±1 *SE* around the mean participant-level ΔLOOIC. Shaded bands in (**B**–**D**, **F**, and **H**), and error bars in (**G**), represent 95% confidence intervals around the fitted values. *SS* Sensation-Seeking.

Critically, because the original task design made expected stimulation and stimulation uncertainty highly correlated (Fig. 2C), we could not reliably estimate their unique or interactive effects in a single model. We therefore compared an expected-stimulation learner (*R* − *θ*) with a stimulation-uncertainty learner (*R* − *ω*), each assigning a bonus to either the learned probability (*Sμ*) or the uncertainty (*Sσ*) values. We compare these Bayesian learning models alongside the original full-knowledge model (Norbury 2015).

Bayesian model comparisons (Fig. 2A) favoured the *R* − *ω* (LOOIC: 6712) over *R* − *θ* (LOOIC: 7673) and original (LOOIC: 7476) alternatives, with lower LOOIC indicating better out-of-sample predictive accuracy. Thus, within the constraints of this design, a model incorporating stimulation uncertainty better captured choices than models based on expected stimulation alone (see Methods for details on model specifications and fitting). Furthermore, we found a similar association between sensation-seeking and the weight on expected stimulation *θ* (Pearson: *t*(65) = 2.52,  *r* = 0.30,  95% *CI* [0.06, 0.50],  *p* = 0.014; Fig. 2B), which replicated the original correlation reported in Norbury 2015 using a model with full knowledge of stimulation probabilities (*t*(65) = 2.67,  *r* = 0.31,  95% *CI* [0.08, 0.52],  *p* = 0.010, Fig. 2C). However, in our winning model, we found a link between the uncertainty weight, *ω*, and sensation-seeking scores (*t*(65) = 2.23,  *r* = 0.27,  95% *CI* [0.03, 0.48],  *p* = 0.029; Fig. 2D). The magnitude of this correlation was similar to that observed in the other two models for expected stimulation, and the uncertainty-weighted learner provided a better predictive fit based on relative LOO-IC. Furthermore, *ω* estimates were negative on average, indicating that most participants treated uncertainty as something to be avoided - consistent with the fact that uncertainty was not informative about reward in this task. However, the positive association between *ω* and sensation-seeking suggests that individuals with higher sensation-seeking showed less uncertainty avoidance. In other words, while most participants discounted uncertain options, those high in sensation-seeking were more willing to sample them, reflecting greater tolerance for stimulation uncertainty in a context where such uncertainty was not directly useful for maximising reward.

To further corroborate this result, we constructed a series of logistic mixed-effects models to assess the impact of *Sμ* and *Sσ* on the probability of choosing an option in a given trial. We investigated how the probability of each choice was influenced by the reward payoff (*Rμ*) and either estimated stimulation probability or stimulation uncertainty, including interactions with sensation-seeking scores. To account for individual variations, we included participant-specific random intercepts. Ranking models by AIC, the best model was again *R* − *ω*, which used stimulation uncertainty and interaction with sensation-seeking scores (AIC: 6394.9; Fig. 2E), compared to the second-best-fitting model, *R* − *θ* (AIC: 7718.2).

We replicated the positive interaction between expected-stimulation and sensation-seeking in our learning model R-*θ* (*β*_*Sμ*×*SS*_ = 0.34,  95% *CI* [0.25, 0.42],  *z* = 7.74,  *p* < 0.001; Fig. 2F), which was similar to that observed in the original full-knowledge model (${\beta }_{{S}_{Identity}\times SS}=0.26,\,95 \% \,CI\,[0.2,0.3],\,z=9.31,\,p < .001$; Fig. 2G). In both cases, high sensation-seekers were more likely to select options associated with stimulation, whereas low sensation-seekers tended to avoid them. In our *winning* model, R-*ω*, there was a strong negative relationship between the probability of selecting some choice A (over alternative B) and the difference in uncertainty (*β*_*Sσ*_ = − 13.56,  95% *CI* [ − 14.4, −12.7],  *z* = − 31.08,  *p* < 0.001), indicating that participants tended to avoid bandits with greater stimulation uncertainty. However, this trend was less pronounced among individuals with high sensation-seeking tendencies (*β*_*Sσ*×*SS*_ = 1.84,  95% *CI* [1.0, 2.7], *z* = 4.26,  *p* < 0.001), who exhibited flatter choice-uncertainty curves (Fig. 2H).

In summary, our reanalysis suggests a different interpretation of the previously reported sensation-seeking effect. Whereas the original analysis framed higher sensation-seeking as greater attraction to stimulation, our learning-based models indicate that choices in this task were better predicted by stimulation uncertainty. Higher sensation-seeking was associated with less negative uncertainty weights, suggesting reduced avoidance of uncertain stimulation. However, because expected stimulation and stimulation uncertainty were highly correlated in the original design, Experiment 1 cannot decisively distinguish these mechanisms or test their interaction. We therefore designed Experiment 2 to reduce this collinearity and to test the unique and joint contributions of expected stimulation and stimulation uncertainty, while also comparing sensation-seeking with impulsivity.

### Experiment 2: Context-dependent uncertainty tolerance

Experiment 2 (Fig. 1B) was a new, preregistered experiment^36^ (See Table S1 for details) in which we removed the collinearity between expected stimulation and uncertainty that had constrained interpretation in Experiment 1 (Fig. 1C). Furthermore, we asked whether sensation-seeking and impulsivity show distinct signatures before turning to model-based analyses.

#### Higher sensation-seeking is associated with broader sampling and lower reward performance

**Fig. 3:**
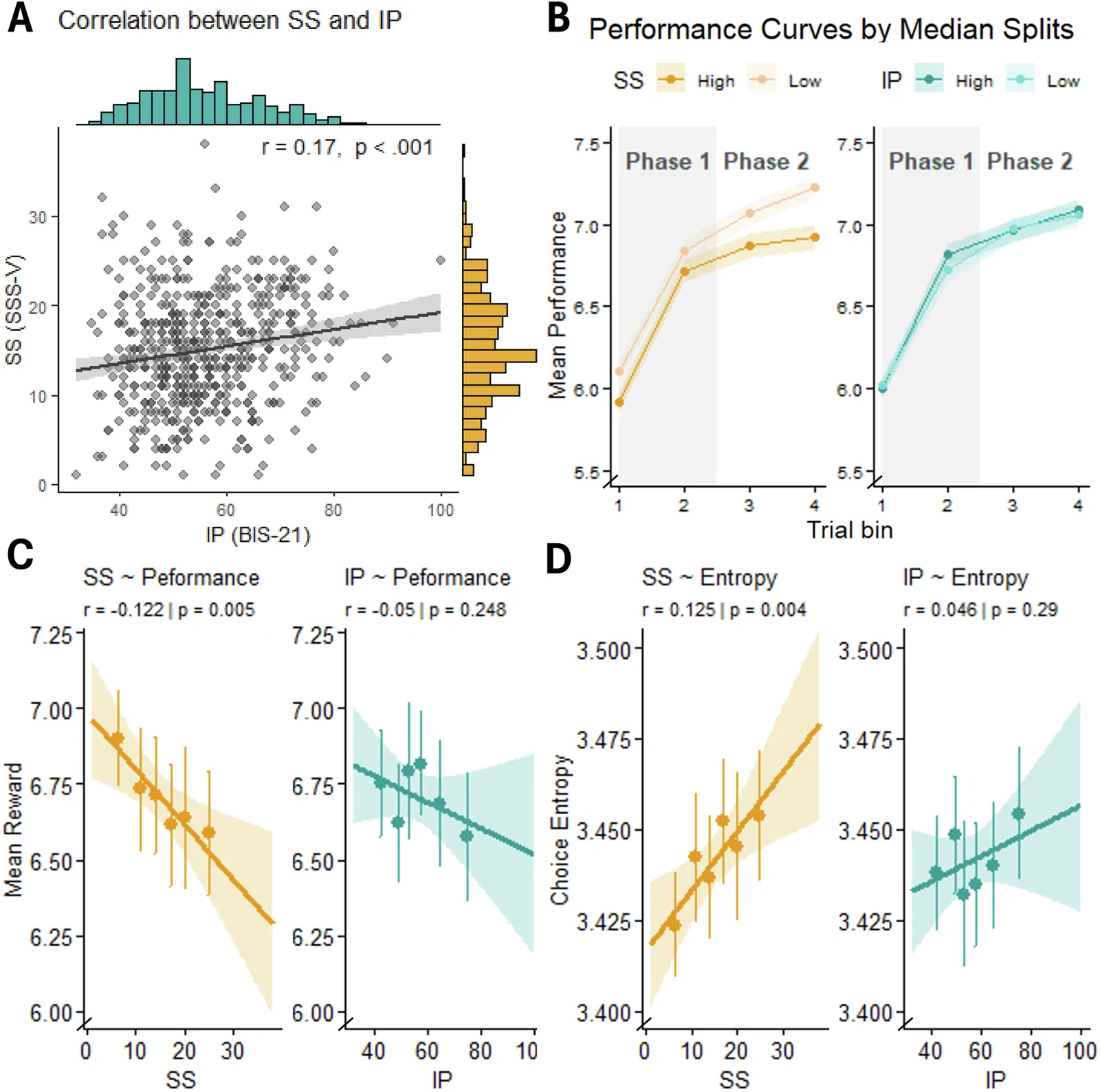
Experiment 2 behavioural results (*n* = 527). **A** Pearson’s correlation between SSS-V and BIS-21 scores (*t*(525) = 3.87, *r* = 0.17, 95% *CI* [0.08, 0.25], *p* = 0.0001215). **B** Learning/performance curves for high- vs low-trait groups (median split). The trial sequence was grouped into bins of 15 trials, corresponding to one full presentation cycle of all object combinations. **C** Correlation of trait and performance, indexed as the mean reward earned across rounds. For SS, we see a negative correlation with performance (*t*(525) = −2.82, *r* = −0.12, 95% *CI* [−0.21, −0.04], *p* = 0.005005). For IP, we found no significant association with performance (*t*(525) = −1.16, *r* = −0.05, 95% *CI* [ −0.14, 0.04], *p* = 0.2484). **D** Correlation of trait and average choice entropy (Shannon entropy of the stimulus-choice distribution) across rounds, with higher values indicating broader sampling. For SS, we observed a positive association with choice entropy (*t*(525) = 2.88, *r* = 0.12, 95% *CI* [0.04, 0.21], *p* = 0.004109). On the other hand, we observed no significant relationship with IP (*t*(525) = 1.06, *r* = 0.05, 95% *CI* [−0.04, 0.13], *p* = 0.2899). All statistical tests were two-sided. No adjustments for multiple comparisons were applied. Shaded bands in (**A**), **C**, and **D**) represent 95% confidence intervals around the fitted regression lines; shaded ribbons in (**B**) represent ±1 SD across participants. SS Sensation-Seeking, IP Impulsivity.

Sensation-seeking and impulsivity were modestly positively correlated in our sample (*t*(525) = 3.87, *r* = 0.17, 95% *CI* [0.08, 0.25], *p* < 0.001; Fig. 3A), but they showed different descriptive relationships with behaviour. We first examined task performance and learning dynamics as the mean reward within 15-trial bins, chosen because each bin contains all pairwise stimulus combinations. Using a regression of performance on trial bin and sensation-seeking, we found a clear effect of learning (*β*_*bin*_ = 0.75, 95% *CI* [0.70, 0.81], *t*(1572) = 26.75, *p* < 0.001) and lower overall reward in higher sensation-seekers (*β*_*SS*_ = −0.11, 95% *CI* [ − 0.19, −0.03], *t*(524) = −2.66, *p* = 0.008; Fig. 3C). Learning curves (Fig. 3B) indicated that this disadvantage was already present in early bins and widened, particularly from the start of phase 2. However, we did not find a significant interaction between SS  × bin (*β*_*SS*×*bin*_ = −0.04, 95% *CI* [−0.09, 0.02], *t*(1572) = −1.35, *p* = 0.18), consistent with a relatively stable performance gap rather than diverging learning curves. Impulsivity groups did not differ reliably in performance in these analyses. Follow-up correlations with overall performance (Fig. 3C) showed a negative relationship with sensation-seeking (*t*(525) = − 2.82, *r* = − 0.12, 95% *CI* [−0.21, −0.04], *p* = 0.005) but again no statistically significant relationship with impulsivity (*t*(525) = −1.16, *r* = −0.05, 95% *CI* [ −0.14, 0.04], *p* = 0.25).

Next, we tested whether sensation-seeking was associated with broader, high-entropy sampling (Fig. 3D).^61–63^. We computed the Shannon entropy over choices, which positively correlated with sensation-seeking scores (*t*(525) = 2.88, *r* = 0.12, 95% *CI* [0.04, 0.21], *p* = 0.004). Thus, high sensation-seekers displayed more diffuse, exploratory choice behaviour. In contrast, impulsivity scores did not significantly correlate with choice entropy (*t*(525) = 1.06, *r* = 0.05, 95% *CI* [−0.04, 0.13], *p* = 0.29).

We next asked whether the sensation-seeking-specific behavioural signatures could be explained by reduced task engagement, boredom, or fatigue. Round-wise performance improved across the session (consistent with practice effects), but high sensation-seekers performed worse overall, and neither sensation-seeking nor impulsivity altered the trajectory of this improvement (Fig. S2, Table S2A). This pattern suggests that accumulating fatigue or disengagement is not the primary driver of sensation-seeking-related performance costs.

To rule out a speed-accuracy account, we examined log reaction times within trial bins (Fig. S3, Table S2B). Reaction times decreased over time, indicating greater efficiency, but sensation-seeking was unrelated to both overall response speed and the rate of change in reaction time. Impulsivity also showed no reliable association with mean reaction times; although there was a small interaction with trial bin, this modulation of reaction time dynamics was not accompanied by any differences in accuracy or reward. Thus, neither trait effect can be reliably explained by a systematic speed-accuracy trade-off.

Finally, we examined omission rates as an index of attention lapses (Fig. S4; Table S2C, D). High sensation-seekers, but not high impulsive participants, missed more trials, and higher omission rates were associated with poorer performance. Crucially, sensation-seeking remained a negative predictor of performance even after statistically controlling for omission rate, indicating that reduced engagement contributes to, but does not fully account for, the sensation-seeking-related performance deficit.

Taken together, higher sensation-seeking is characterised by broader, exploratory sampling (higher entropy) and a modest but reliable performance cost that cannot be attributed solely to fatigue, global disengagement, or speed-accuracy trade-offs, even though high sensation-seekers show slightly elevated omission rates. In contrast, impulsivity does not show the same profile. These behavioural results thus provide a focused, trait-specific foundation for the subsequent model-based analyses.

#### Sensation-seeking and impulsivity show opposing modulation of stimulation uncertainty

**Fig. 4:**
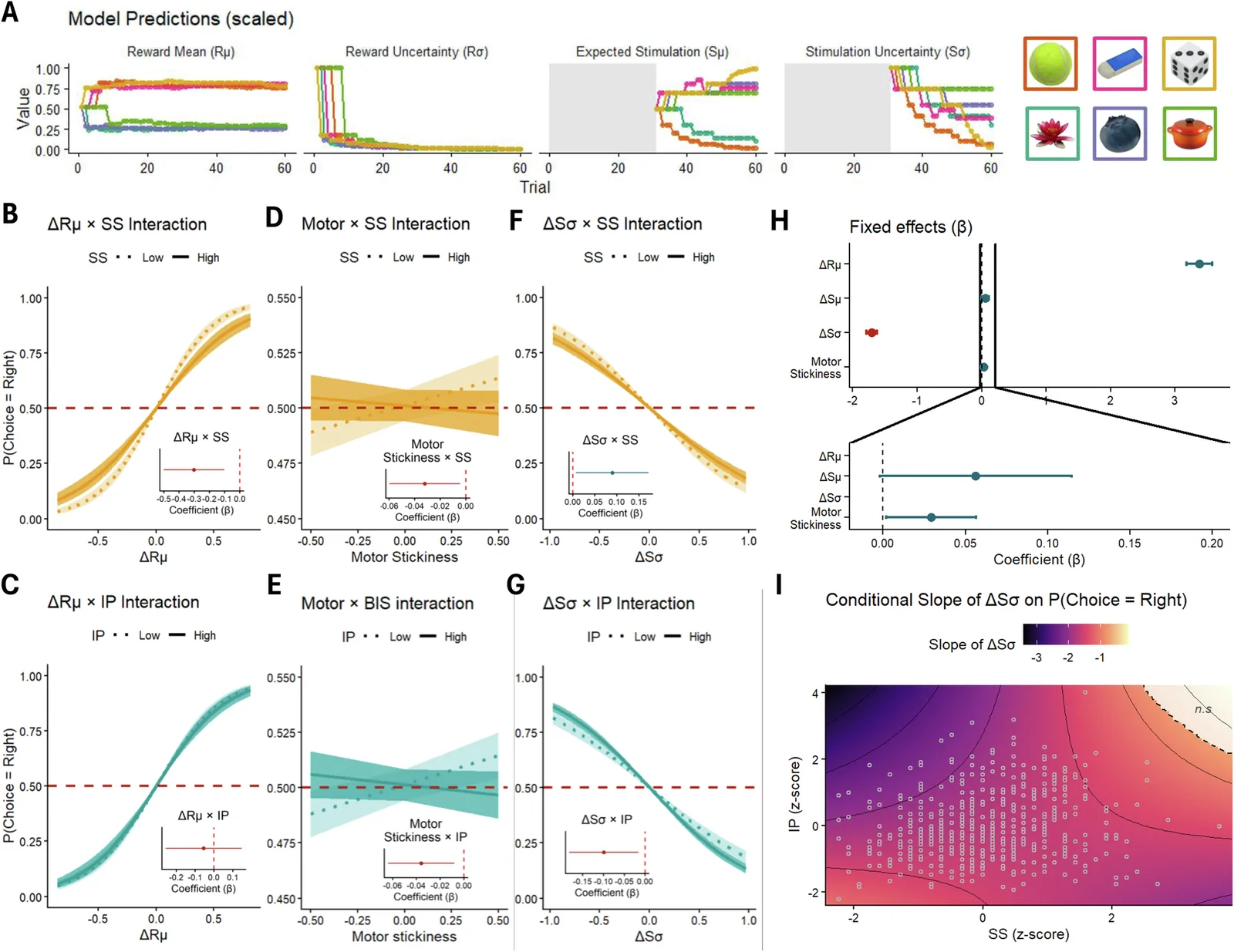
Model predictors, trait interactions, and fixed-effect estimates for the choice model (*n* = 527). **A** Scaled time courses of model-derived predictors across a block: reward mean (*Rμ*), reward uncertainty (*Rσ*), stimulation mean (*Sμ*), and stimulation uncertainty (*Sσ*) for one example participant. **B**, **C** Predicted *P*(choice = Right) as a function of Δ*Rμ* at  ± 2 SD of SS and IP, respectively. The Δ*Rμ* × SS interaction was significant (*β* = −0.30, 95% *CI* [−0.50, −0.10], *z* = − 2.95, *p* = 0.00316), whereas the Δ*Rμ* × IP interaction was not significant (*β* = −0.054, 95% *CI* [−0.257, 0.149], *z* = − 0.52, *p* = 0.60084). The red dashed line marks *P* = 0.5. **D**, **E** Motor stickiness  × SS (*β* = − 0.032, 95% *CI* [−0.060, −0.004], *z* = −2.27, *p* = 0.02295) and motor stickiness  × IP (*β* = −0.036, 95% *CI* [−0.064, −0.008], *z* = −2.52, *p* = 0.01187) effects on choice. **F**, **G**Δ*Sσ* × SS (*β* = 0.090, 95% *CI* [0.007, 0.172], *z* = 2.13, *p* = 0.03288) and Δ*Sσ* × IP (*β* = − 0.099, 95% *CI* [−0.182, −0.016], *z* = −2.34, *p* = 0.01944) effects on choice. The two interaction coefficients differed significantly (Δ*β* = 0.189, 95% *CI* [0.061, 0.317], *χ*^2^(1) = 8.33, *p* = 0.003904). **H** Forest plot of fixed effects for the full GLMM. **I** Conditional effect (slope) of Δ*Sσ* on *P*(choice = Right) as a function of SS and IP (Johnson–Neyman-style surface). The Δ*Sσ* × SS × IP interaction was significant (*β* = 0.103, 95% *CI* [0.023, 0.184], *z* = 2.52, *p* = 0.01164). All statistical tests were two-sided. No adjustments for multiple comparisons were applied. Shaded ribbons in (**B**–**G**) represent 95% confidence intervals around model-predicted probabilities. Points and error bars in the insets of **B**–**G**, **H** represent fixed-effect estimates (*β*) and their 95% confidence intervals, respectively. *Rμ* reward mean, *Rσ* reward uncertainty, *Sμ* stimulation mean, *Sσ* = stimulation uncertainty, Δ difference between right and left option predictor values, SS sensation-seeking, IP impulsivity.

We then asked what computational features underpinned the higher choice variability observed in high sensation-seekers. Using our learning models of reward (Bayesian Mean Tracker; BMT) and stimulation (Bayesian Learner Beta-Binomial; BLBB), we derived trial-by-trial predictors of reward mean (R*μ*), reward uncertainty (R*σ*) (Fig. 4A, left), expected stimulation (S*μ*) and stimulation uncertainty (S*σ*) (Fig. 4A, right). R*σ* was estimated for visualisation but not included in the final choice rule for reasons described below.

We first compared two stickiness formulations using AIC scores (Table S3): a model with motor perseveration (previous response side; *AIC* = 141098), which outperformed a stimulus perseveration model (previous chosen bandit; *AIC* = 141135.4) that we preregistered (see Table S1 for preregistered analyses). Therefore, subsequent analyses used the better motor perseveration model, which revealed a significant effect of motor perseveration (*β*_*sticky*_ = 0.029, 95% *CI* [0.002, 0.056], *z* = 2.11, *p* = 0.035).

We then entered these trial-level predictors into a mixed-effects logistic regression (Fig. 4B–H). Reward uncertainty (R*σ*) was not included in the decision rule because the full feedback paradigm has previously been shown to substantially reduce epistemic choice^64^. In addition, reward variability was intentionally kept low, and excluding R*σ* provided a more parsimonious model specification. Choices were modelled as a function of the difference (denoted Δ) in expected reward (ΔR*μ*), expected stimulation (ΔS*μ*), stimulation uncertainty (ΔS*σ*), and a motor stickiness term, with participant-specific random intercepts and random slopes for ΔR*μ*. As preregistered, participant choices reliably tracked value and avoided uncertainty: larger Δ*Rμ* increased the odds of choosing right (*β*_*Rμ*_ = 3.38, 95% *CI* [3.18, 3.58], *z* = 33.20, *p* < 0.001), whereas larger Δ*Sσ* decreased it (*β*_*Sσ*_ = − 1.70, 95% *CI* [−1.78, −1.62], *z* = − 41.68, *p* < 0.001). The stimulation mean was trending towards significance (*β*_*Sμ*_ = 0.056, 95% *CI* [−0.002, 0.114], *z* = 1.89, *p* = 0.058) in the expected direction (Fig. 4H).

We then tested whether sensation-seeking and impulsivity traits modulate these computational signals by interacting with Δ*Rμ* and Δ*Sσ*, while also including the motor stickiness term (with all two- and three-way terms). Mirroring behavioural patterns, sensation-seeking reduced reward sensitivity (*β*_*SS*__×__*Rμ*_ = −0.30, 95% *CI* [ −0.50, −0.10], *z* = −2.95, *p* = 0.003; Fig. 4B), but not for impulsivity (*β*_*IP*__×__*Rμ*_ = −0.054, 95% *CI* [−0.257, 0.149], *z* = −0.52, *p* = 0.601; Fig. 4C). Both traits also modulated motor perseveration in the same direction (*β*_*SS*__×__*Sticky*_ = −0.032, 95% *CI* [−0.060, −0.004], *z* = −2.27, *p* = 0.023; *β*_*IP*__×__*Sticky*_ = −0.036, 95% *CI* [−0.064, −0.008], *z* = − 2.52, *p* = 0.012), suggesting that higher sensation-seeking and higher impulsivity each dampen value-free side-repetition once reward value and stimulation uncertainty are accounted for (Fig. 4D, E).

Crucially, and consistent with Experiment 1 and our preregistration, sensation-seeking was associated with less uncertainty aversion: higher sensation-seeking weakened the negative impact of stimulation uncertainty on choice (*β*_*SS*×*Sσ*_ = 0.090, 95% *CI* [0.007, 0.172], *z* = 2.13, *p* = 0.033; Fig. 4F). In contrast, impulsivity showed the opposite pattern, being associated with greater avoidance of stimulation uncertainty (*β*_*IP*__×__*Sσ*_ = −0.099, 95% *CI* [−0.182, −0.016], *z* = −2.34, *p* = 0.019; Fig. 4G). To directly test whether sensation-seeking and impulsivity differed in their modulation of stimulation uncertainty, we compared the *β*_*SS*__×__*Sσ*_ and *β*_*IP*__×__*Sσ*_ coefficients within the same mixed-effects model. This contrast was significant (Δ*β* = 0.189, 95% *CI* [0.061, 0.317], *χ*^2^(1) = 8.33, *p* = 0.004). Higher sensation-seeking weakened avoidance of uncertain stimulation, whereas higher impulsivity strengthened it, indicating that the two traits had opposing and statistically distinguishable associations with stimulation uncertainty.

We also observed a three-way interaction between Δ*Sσ* and both sensation-seeking and impulsivity traits (*β*_*SS*×*IP*__×__*Sσ*_ = 0.103, 95% *CI* [0.023, 0.184], *z* = 2.52, *p* = 0.012). To interpret this three-way interaction, we plotted the conditional slopes of Δ*Sσ* across the observed sensation-seeking and impulsivity space (Fig. 4I). Here, we observe how the Δ*Sσ* slope becomes less negative as sensation-seeking increases, but more negative as impulsivity increases. The strongest levels of uncertainty aversion are thus observed at low sensation-seeking and high impulsivity (top-left corner), while for individuals high in both sensation-seeking and impulsivity (top-right corner), the effect of Δ*Sσ* on choice is close to zero and not reliably different from zero, indicating that uncertainty no longer systematically deters choice in this subgroup.

#### Expected stimulation and uncertainty jointly distinguish sensation-seeking from impulsivity

**Fig. 5:**
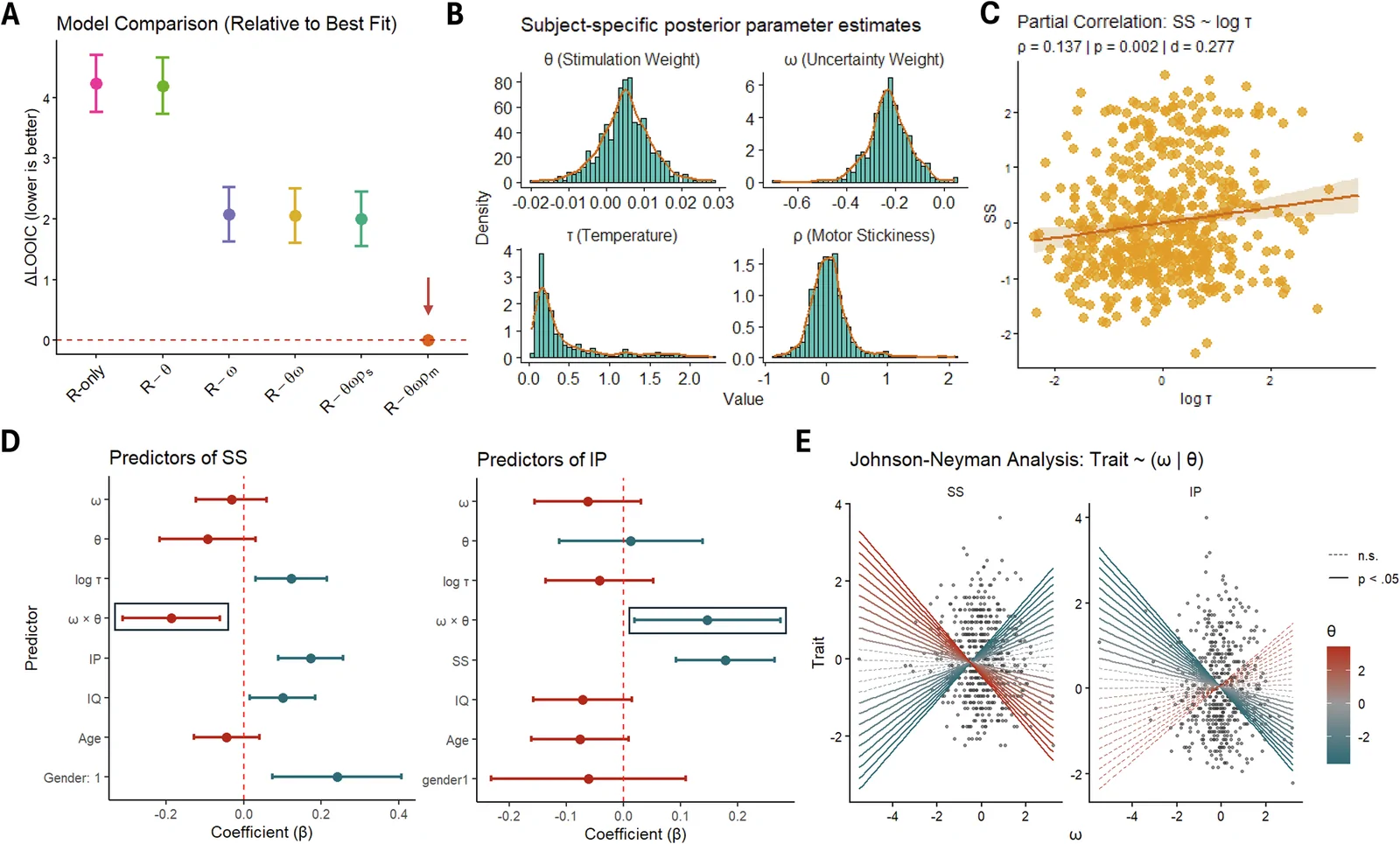
Hierarchical Bayesian Modelling results (*n* = 527). **A** Model comparison with reduced versions of our full model, as well as an alternative version of stickiness, which is based on stimulus identity. We find that our full model specification, which includes a motor stickiness bonus, best accounts for individual differences in choice behaviour. **B** Distribution of subject-specific posterior mean parameter estimates. **C** Partial correlation of sensation seeking (SS) with temperature $\log (\tau )$ after controlling for impulsivity (IP), age, IQ and gender. We observed a positive association (*t*(521) = 3.16, ${r}_{SS-\log (\tau )}=0.137$, 95% *CI* [0.052, 0.220], *p* = 0.002), suggesting that higher SS is associated with a global increase in choice stochasticity. **D** Regression coefficients predicting traits as interactions between computational parameters controlling for various correlates. We observe that SS is significantly predicted by increasing temperature (*β* = 0.124, 95% *CI* [0.030, 0.218], *t*(514) = 2.59, *p* = 0.00981), as well as a *negative* interaction between expected stimulation weighting *θ* and uncertainty *ω* (*β* = −0.187, 95% *CI* [ −0.312, −0.062], *t*(514) = −2.94, *p* = .00347; boxed). Conversely, IP is predicted by a *positive* interaction between *θ* and *ω* (*β* = 0.147, 95% *CI* [0.020, 0.275], *t*(514) = 2.27, *p* = .0237). This highlights distinct parameter--trait profiles, with the *θ* × *ω* association differing significantly between SS and IP (Δ*β* = −0.284, 95% *CI* [ −0.431, −0.137], *z* = −3.78, *p* < 0.001). All predictors were z-scored before analysis. **E** Johnson–Neyman fanplots of the *θ* × *ω* interaction. Conditional slopes of *ω* against traits within the observed range of moderator *θ*. Bold solid lines indicate statistically significant relationships (*p* < 0.05), while dotted lines did not reach significance. Dots are raw subject points. We observed that IP is associated with a decrease in *ω* at below-average values of *θ* (*θ*_*z*_≤ −0.27). Conversely, SS is associated with increasing values of *ω* at below-average values of *θ* (*θ*_*z*_≤ −1.01), but decreasing *ω* at above-average values of *θ* (*θ*_*z*_ ≥ 0.38). Error bars in (**A**) represent  ±1 SE around the mean participant-level ΔLOOIC. The shaded band in (**C**) represents the 95% confidence interval around the fitted regression line. Error bars in (**D**) represent 95% confidence intervals around coefficient estimates. All statistical tests were two-sided. No adjustments for multiple comparisons were applied. SS sensation seeking, IP impulsivity.

We then fit a hierarchical, generative Bayesian choice model that decomposes trial-wise decisions into *Rμ*, *Sμ*, *Sσ*, and a motor-stickiness bonus. This yielded subject-level parameters *θ* (bonus on *Sμ*), *ω* (bonus on *Sσ*), *ρ* (stickiness bonus), and *τ* (softmax temperature; choice stochasticity). Model comparison indicated that the full specification (reward, expected stimulation, stimulation uncertainty, and motor stickiness) outperformed reduced alternatives (Fig. 5A; see Methods). Posterior predictive checks indicated that the winning model reproduced the main trial-level choice patterns (Fig. S9). Simulated choices closely tracked the observed increase in choosing the right option as *Rμ* increased and the observed decrease as *Sσ* increased. Expected stimulation *Sμ* showed little aggregate association with choice, suggesting that its influence was not expressed as a uniform population-level effect but rather through individual differences or interactions with uncertainty.

Using subject-specific posterior estimates (Fig. 5B), we computed partial Pearson correlations between traits and model parameters while controlling for impulsivity, age, IQ, and gender. Contrary to our preregistered prediction, sensation-seeking was not significantly associated with *ω* (*t*(521) = −1.82, *r*_*SS*−*ω*_ = −0.079, 95% *CI* [ −0.164, 0.006], *p* = 0.070), but positively correlated with $\log (\tau )$ (*t*(521) = 3.16, ${r}_{SS-\log (\tau )}=0.137$, 95% *CI* [0.052, 0.220], *p* = 0.002; Fig. 5C), consistent with higher choice randomness among high sensation-seekers. None of the zero-order or partial associations between impulsivity and individual model parameters reached statistical significance (see Figs. S11, S12).

Motivated by the trial-level moderation pattern (Fig. 4I), we conducted exploratory follow-up parameter-level analyses testing whether sensation-seeking and impulsivity were associated with *combinations* of computational parameters. To achieve this, we regressed our model parameters and their interactions, adjusting for covariates, on our traits of interest (Fig. 5D, Fig. S13).

We observed that sensation-seeking remained positively associated with choice stochasticity (*β* = 0.124, 95% *CI* [0.030, 0.218], *t*(514) = 2.59, *p* = .00981). More critically, we observed a *negativeθ* × *ω* interaction (*β* = −0.187, 95% *CI* [ −0.312, −0.062], *t*(514) = −2.94, *p* = 0.00347). A Johnson–Neyman^65,66^ analysis (Fig. 5E, S14) into the interaction restricted within our observed moderator range ( − 3.67≤*θ*_*z*_ ≤ 3.46) showed that the conditional slope of *ω* on sensation-seeking was *positive* when *θ*_*z*_ ≤ −1.01 but *negative* when *θ*_*z*_ ≥ 0.38. Because more positive *ω* values indicate weaker avoidance of stimulation uncertainty, this pattern suggests that higher sensation-seeking was associated with greater uncertainty tolerance when expected-stimulation weighting was low, but with greater uncertainty avoidance when expected-stimulation weighting was high.

We then applied the same model to impulsivity. Although no main effects of the HB parameters emerged as statistically significant, there was a *positiveθ* × *ω* interaction (*β* = 0.147, 95% *CI* [0.020, 0.275], *t*(514) = 2.27, *p* = .0237); Johnson–Neyman analysis indicated that the conditional slope of *ω* on impulsivity was significantly negative when expected-stimulation weighting was low (*θ*_*z*_≤ −0.27).

To directly compare the parameter-level associations between sensation-seeking and impulsivity, we fitted a joint path model^67,68^ that predicted both traits from the same set of computational parameters and covariates, while allowing their residual covariances to be estimated freely. The critical contrast showed that the *θ* × *ω* association differed significantly between sensation-seeking and impulsivity (Δ*β* = −0.284, 95% *CI* [−0.431, −0.137], *z* = −3.78, *p* < 0.001). Descriptively, this interaction was negative for sensation-seeking but positive for impulsivity, indicating that expected stimulation and uncertainty weighting were combined differently across the two traits.

Because the subjective experience of the stimulation could contribute to expected-stimulation weighting, we conducted exploratory analyses using participants’ valence and arousal ratings (Fig. S16). Participants rated the stimulation as pleasant and moderately arousing, with both valence (*M* = 78.30, 95% *CI* [76.56, 80.04], *t*(526) = 32.03, *p* < 0.001) and arousal (*M* = 64.97, 95% *CI* [62.78, 67.17], *t*(526) = 13.37, *p* < .001) significantly above the neutral midpoint. In covariate-adjusted regressions, sensation-seeking was not significantly associated with valence ratings (*β* = −0.019, 95% *CI* [−0.105, 0.067], *t*(521) = −0.44, *p* = 0.664), but was positively associated with arousal ratings (*β* = 0.099, 95% *CI* [0.013, 0.185], *t*(521) = 2.26, *p* = 0.024). By contrast, impulsivity was associated with both lower valence (*β* = −0.179, 95% *CI* [ −0.264, −0.093], *t*(521) = −4.10, *p* < 0.0001) and lower arousal (*β* = −0.158, 95% *CI* [ −0.243, − 0.072], *t*(521) = −3.63, *p* < 0.001). Importantly, neither valence nor arousal significantly predicted expected-stimulation weighting (*θ*; valence: *β* = 0.034, 95% *CI* [−0.065, 0.132], *t*(519) = 0.67, *p* = 0.501; arousal: *β* = −0.053, 95% *CI* [−0.151, 0.045], *t*(519) = − 1.05, *p* = . 292) or uncertainty weighting (*ω*; valence: *β* = −0.074, 95% *CI* [−0.170, 0.023], *t*(519) = −1.49, *p* = 0.137; arousal: *β* = −0.012, 95% *CI* [−0.109, 0.085], *t*(519) = −0.25, *p* = 0.806), after adjusting for sensation-seeking, impulsivity, age, IQ, and gender. Repeating the joint trait path model with valence and arousal included as covariates yielded the same critical result — the *θ* × *ω* association differed significantly between sensation-seeking and impulsivity (Δ*β* = −0.256, 95% *CI* [−0.405, −0.107], *z* = −3.37, *p* = .001). Thus, the distinct parameter–trait profiles for sensation-seeking and impulsivity were not explained by subjective ratings of stimulation valence or arousal.

Together, these results suggest that sensation-seeking and impulsivity have distinct parameter-trait profiles, driven by an interplay between expected stimulation and uncertainty weighting. Intuitively, the association between sensation-seeking and uncertainty weighting is *context-dependent*: higher sensation-seeking was associated with greater uncertainty tolerance when expected-stimulation weighting was low, but with greater uncertainty avoidance when expected-stimulation weighting was high. For impulsivity, the pattern was more restricted, with higher impulsivity primarily associated with stronger uncertainty avoidance when expected-stimulation weighting was low. These results suggest that sensation-seeking and impulsivity do not merely differ in overall risk propensity or choice stochasticity, but in how stimulation value and uncertainty jointly shape choice.

## Discussion

Sensation-seeking is often framed as a tendency to pursue novel and stimulating experiences. Yet stimulating options are also uncertain. Choices toward stimulating options may reflect attraction to expected stimulation, tolerance of uncertainty, or the way these quantities are combined. Across two experiments, we separated expected stimulation from stimulation uncertainty and asked whether these decision variables explain sensation-seeking-related choice, and whether this profile differs from that of impulsivity. The central finding was that sensation-seeking was not reducible to expected-stimulation seeking or to a general preference for uncertainty. Instead, sensation-seeking-related choice was characterised by two complementary signatures: greater overall choice stochasticity and a context-dependent relationship between expected-stimulation weighting and uncertainty weighting. This profile differed from impulsivity, suggesting that sensation-seeking and impulsivity do not differ only in overall risk propensity or decision noise, but also in how stimulation value and uncertainty enter the choice rule.

Experiment 1 first suggested that stimulation uncertainty may be important for understanding prior sensation-seeking effects. Participants with higher sensation-seeking scores were less deterred by uncertainty about stimulation and more often sampled options when stimulation probability was poorly specified. However, expected stimulation and stimulation uncertainty were correlated in that design, limiting the ability to distinguish whether sensation-seeking was related to stimulation value, uncertainty tolerance, or both. Experiment 2, therefore, used a preregistered task that decorrelated expected stimulation and stimulation uncertainty^36^. This revealed a convergent but more nuanced pattern. Behaviourally, higher sensation-seeking was associated with broader sampling and lower reward performance. At the trial level, higher sensation-seeking weakened avoidance of stimulation uncertainty, whereas higher impulsivity strengthened it, with a direct sensation-seeking–impulsivity contrast supporting this dissociation.

Our hierarchical modelling approach clarified this dissociation at the parameter level by separating global choice stochasticity from feature-specific uncertainty weighting. Higher sensation-seeking was associated with greater choice stochasticity, indexed by higher temperature (*τ*), linking the descriptive entropy effect to a generative account of choice. In other words, sensation-seekers sampled more broadly partly because their choices were less consistently governed by the modelled value difference. However, stochasticity alone did not capture the full sensation-seeking profile. Broader sampling can arise from several mechanisms, including a more stochastic choice policy, greater tolerance of uncertain options, or stronger weighting of expected stimulation. These mechanisms can look similar behaviourally but imply different choice computations. In the present data, high sensation-seekers were not simply random or inattentive, but rather showed a structured interaction between expected-stimulation weighting (*θ*) and uncertainty weighting (*ω*). When expected-stimulation weighting was low, higher sensation-seeking was associated with greater tolerance of stimulation uncertainty; when expected-stimulation weighting was high, higher sensation-seeking was associated with greater avoidance of uncertain stimulation. Thus, sensation-seeking was linked both to how consistently participants followed the value structure of the task and to how stimulation value shaped whether uncertainty was tolerated or avoided.

These findings refine how sensation-seeking should be understood in relation to exploration. In theories of directed exploration and curiosity, uncertain options may be sampled because they provide useful information for future choice^24,25^. The present task isolated a different case. Because stimulation probabilities and reward levels were generated independently in our task design, neither receiving stimulation nor learning about its probability could help participants identify higher-reward options. Accordingly, the broader sampling associated with sensation-seeking should not be interpreted as adaptive information-seeking in this task, but rather described as uncertainty-tolerant sampling in a context where such sampling carries a reward cost. This distinction may help reconcile mixed findings in the broader literature, where sensation-seeking has been linked to both beneficial outcomes, such as novelty seeking, exploration, and well-being, and harmful outcomes, such as substance use and risky behaviour^4,7,8,10–16,69,70^. The present findings suggest that these divergent associations may partly depend on how stimulation value and uncertainty are jointly weighted in a given environment. When uncertainty provides information, opportunity, or long-term value, sensation-seeking-related sampling may support curiosity-driven exploration; when uncertainty is non-instrumental or competes with more reliable goals, the same tendency may reduce reward optimisation. Thus, while our findings do not show that sensation-seeking is adaptive or maladaptive in general, they identify a candidate decision mechanism through which sensation-seeking may have different consequences across contexts.

The comparison with impulsivity further sharpens this account. Although sensation-seeking and impulsivity are both associated with risk-taking, they did not map onto the same computational profile. Impulsivity was not reliably associated with greater choice stochasticity or with the broader-sampling and lower-performance pattern observed for sensation-seeking. Instead, higher impulsivity was associated with stronger avoidance of stimulation uncertainty, particularly when expected-stimulation weighting was low. This contrasts with prior work linking impulsivity to value-free randomness in reward-based or affectively neutral exploration tasks^71–73^. One possibility is that the behavioural expression of impulsivity depends on the domain of uncertainty. When uncertainty concerns reward or neutral information, impulsivity may manifest as noisier or less goal-directed sampling. When uncertainty arises about whether stimulation will occur, especially if it is affectively salient or potentially aversive, uncertainty avoidance may become more pronounced. Because the present task measured but did not manipulate affect, this interpretation remains a hypothesis for future work.

Together, these findings point to a computational dissociation between sensation-seeking and impulsivity. Sensation-seeking was associated with a broader, more stochastic choice policy, as well as with a structured interaction between expected-stimulation weighting and uncertainty weighting. This suggests that sensation-seeking shapes exploratory choice through two separable routes. First, by increasing the breadth of sampling overall, and by changing when uncertain stimulation is tolerated versus avoided. Impulsivity showed a significantly different profile, being more closely associated with avoidance of stimulation uncertainty than with stochastic sampling. This therefore shows that sensation-seeking and impulsivity are not simply distinct constructs, but also specifies that this distinction is expressed in the computations that govern choice.

### Limitations and future directions

Several limitations should be considered. First, Experiment 1 provided useful evidence for an uncertainty-based reinterpretation of prior sensation-seeking effects, but the original design correlated expected stimulation and stimulation uncertainty. The reanalysis, therefore, motivated, rather than conclusively established, the need to separate these components. Experiment 2 addressed this limitation by decorrelating expected stimulation from stimulation uncertainty, but did so using a simplified stimulus. This simplification provides a methodological strength as real-world sensation-seeking contexts combine stimulation value, uncertainty, reward, affect, social meaning, and risk, making it difficult to identify which component drives choice. By using a controlled artificial analogue, we were able to cleanly separate our computational quantities of interest and isolate their effects on choice. The trade-off is that the present task prioritises computational identifiability over ecological richness, and future work should test whether the same mechanisms generalise to more clinically and motivationally salient forms of sensation-seeking, such as substance use, gambling, social risk-taking, or high-intensity physical activities.

Second, our results suggest that subjective affective responses to stimulation may be important. Participants rated the visual stimulation as pleasant and moderately arousing, and these ratings were included in robustness analyses. However, valence and arousal were measured rather than experimentally manipulated. This matters because expected-stimulation weighting (*θ*) may depend on how pleasant, arousing, or goal-relevant the stimulation is for a given individual. Future studies should manipulate stimulation value directly, for example, by varying affective valence, arousal, modality, or goal relevance^74^, to test whether sensation-seeking shifts along the *θ* − *ω* surface as stimulation becomes more or less subjectively valuable.

Third, the present interpretation depends on computational modelling choices. The winning hierarchical model separated reward, expected stimulation, stimulation uncertainty, and value-free stickiness, and posterior predictive checks indicated that it captured key trial-level and trait-binned behavioural patterns. Nevertheless, temperature-like parameters can absorb multiple sources of unexplained variability, including stochastic exploration, lapses, imperfect learning, or unmodelled decision variables^75^. We therefore interpret the association between sensation-seeking and choice stochasticity as evidence for a broader, less deterministic choice policy rather than as a pure measure of random exploration.

Finally, both experiments used adult samples. The findings, therefore, do not permit conclusions about developmental change, even though they may inform developmental theories of risk-taking. Developmental accounts often distinguish sensation-seeking from impulsivity, proposing that these traits follow different trajectories and reflect partially distinct neurocognitive systems^28^. The present results add a computational hypothesis to this distinction, in which sensation-seeking and impulsivity may differ not only in their associations with risk-taking outcomes, but also in how they weigh stimulation and uncertainty during choice. Testing this hypothesis will require developmental samples and tasks that separate expected stimulation, uncertainty, and the usefulness of uncertainty across age.

Despite these limitations, the present framework generates testable predictions for future work. If expected-stimulation weighting changes how uncertainty is treated, then increasing the subjective value or salience of stimulation should shift high sensation-seekers toward more selective pursuit of predictable stimulation. Gambling environments provide one possible domain in which this interaction could be tested. Salient audiovisual cues in slot-machine play can increase immersion and alter reward-related choice^76–78^, while features such as losses disguised as wins and near-misses can elevate arousal and promote persistence despite negative expected value^79–84^. In terms of the present framework, such environments may increase the subjective value of stimulation while making low-value outcomes feel more engaging, potentially biasing high sensation-seekers toward persistent engagement with predictable but costly options. This interpretation remains speculative, as the present task did not experimentally manipulate these quantities. Nevertheless, it suggests reducing cue salience, for example by attenuating audiovisual intensity or neutralising win-like animations, should reduce expected-stimulation weighting, whereas increasing usable information, for example through clearer odds or volatility displays, should make uncertainty more interpretable. Future work could test whether these manipulations weaken persistent engagement among high sensation-seekers by shifting the balance between stimulation value and uncertainty weighting.

## Supplementary information

Supplementary Material

Transparent Peer Review file

## Data Availability

Experiment 1 comprised secondary analyses of datasets originally collected by Norbury et al. (2015)^17^. The data were provided by the original authors for secondary analysis. As the present authors do not have authorisation to redistribute these datasets, the participant-level data and associated source data for Experiment 1 are not publicly available from this study, and requests for access should be directed to the original data holders. Source data and de-identified participant-level data for Experiment 2 are available at https://zenodo.org/records/21520478^85^.

## Code availability

Custom analysis and computational modelling code for Experiments 1 and 2 to fully reproduce the main findings, figures and supplementary material are publicly available at https://zenodo.org/records/21520478^85^.
