# Supplementary material for: Stimulation uncertainty reveals distinct mechanisms of sensation seeking and impulsivity
Source: Commun Psychol. 2026 Oct 23;4:141. doi: 10.1038/s44271-026-00525-5 (PMC13694268; doi:10.1038/s44271-026-00525-5)
Supplement: Supplementary file 1 [file 44271_2026_525_MOESM1_ESM.pdf]

Supplementary Information for:

Stimulation Uncertainty Reveals Distinct Mechanisms of Sensation Seeking and Impulsivity

Ern Wong[1,2,*], **Tobias U. Hauser**[3,4,5], **Aswath Chandrasekaran**[2], **Pietro Pietrini**[1], and **Charley M. Wu**[6,7]

[1]MoMiLab, IMT School for Advanced Studies Lucca, Lucca, Italy
[2]Human and Machine Cognition Lab, Technische Universität Darmstadt, Darmstadt, Germany
[3]Department of Psychiatry and Psychotherapy, Faculty of Medicine, University of Tübingen, Tübingen, Germany
[4]German Centre for Mental Health (DZPG), Tübingen, Germany
[5]Max Planck UCL Centre for Computational Psychiatry and Ageing Research, University College London, London, UK
[6]Center for Cognitive Science, Technische Universität Darmstadt, Darmstadt, Germany
[7]Hessian AI, Darmstadt, Germany
[*]Correspondence: ern.wong@imtlucca.it

Preregistration Details

Table S1. Preregistered hypotheses, analysis plans, and deviations for Experiment 2. The preregistration was submitted on 14 June 2025 prior to data collection. "Partially supported" indicates that part of the preregistered prediction was supported but that either not all predicted effects were observed or the implemented analysis differed from the preregistered specification. Analyses introduced after preregistration are identified as exploratory.

Item	Preregistered prediction / plan	Manuscript status / deviation
**Preregistered hypotheses**		
H1a	Participants would use trial-wise reward mean ($R\mu$), stimulation mean ($S\mu$), stimulation uncertainty ($S\sigma$), and a history-weighted perseveration/familiarity term to guide choices.	**Partially supported.** Choices reliably tracked $R\mu$ and $S\sigma$, while the population-level effect of $S\mu$ did not reach statistical significance. The final model included motor stickiness rather than the preregistered familiarity-based perseveration term, based on model comparison.
H1b	Subjective arousal ratings of the stimulation, more than valence ratings, would modulate stimulation-related regressors. Stimulation-related predictors were planned to be multiplicatively weighted by standardised arousal ratings.	**Modified/exploratory.** Subjective arousal was assessed retrospectively at the end of each round, after the choices it was intended to moderate. We therefore considered multiplicatively weighting trial-wise stimulation regressors by these post-round ratings to be methodologically suboptimal, as this would use a post-choice measure to parameterise preceding trial-level decisions and would conflate the stimulation signal with subjective arousal. We therefore did not implement the preregistered arousal-weighted regressors. Instead, valence and arousal were examined separately in exploratory covariate-adjusted analyses to test whether subjective responses to stimulation accounted for the observed trait–parameter associations.
H1c	Impulsivity would be associated with lower reward sensitivity.	**Not supported.** Impulsivity did not reliably modulate reward sensitivity in the primary mixed-effects choice model.
H1d	Sensation-seeking and impulsivity would interact in their association with reward sensitivity, such that impulsive sensation-seeking would be associated with lower reward sensitivity, whereas high sensation-seeking combined with low impulsivity would be associated with greater reward sensitivity.	**Not supported.** The preregistered interaction between sensation-seeking and impulsivity on reward sensitivity was not supported.
H1e	Sensation-seeking would interact with stimulation uncertainty ($S\sigma$), weighted by subjective arousal, such that individuals lower in sensation-seeking would show stronger aversion to stimulation uncertainty.	**Partially supported.** Higher sensation-seeking was associated with weaker avoidance of stimulation uncertainty, consistent with the predicted direction. However, stimulation uncertainty was not multiplicatively weighted by arousal for reasons described above in H1b.
H2a	A hierarchical Bayesian model incorporating reward, stimulation mean, stimulation uncertainty, and a history-weighted familiarity/perseveration bias would provide the best fit to choice behaviour.	**Partially supported.** The best-fitting hierarchical Bayesian model included reward, stimulation mean, stimulation uncertainty, and motor stickiness. Motor rather than stimulus/familiarity-based stickiness provided the better-fitting perseveration term.
H2b	The stimulation-uncertainty parameter (ω) would be positively associated with sensation-seeking after controlling for arousal and impulsivity (BIS).	**Not supported as preregistered.** A reliable positive association between ω and sensation-seeking was not observed. Subsequent analyses examining interactions between expected-stimulation weighting (θ) and uncertainty weighting (ω) were exploratory.

Continued on next page

Table S1 continued from previous page

Item	Preregistered prediction / plan	Manuscript status / deviation
H2c	The familiarity-bias parameter (η) would be negatively associated with sensation-seeking after controlling for arousal and impulsivity (BIS).	**Not supported.** The preregistered familiarity-bias analysis was conducted using the stimulus/familiarity-stickiness parameter from the corresponding hierarchical model. Contrary to the preregistered prediction, familiarity bias was not significantly associated with sensation seeking after controlling for impulsivity, age, IQ, gender, arousal, and valence, $r(519) = 0.065$, 95% CI $[-0.021, 0.151]$, $p = 0.136$. The final primary model retained motor rather than stimulus/familiarity stickiness because it provided a better predictive fit. Thus, this preregistered analysis is reported in the Supplementary Information.
**Preregistered exclusion criterion**		
Exclusion criterion	Participants performing below chance, defined as achieving less than 50% of the maximum possible score on the experimental task, would be excluded.	**Modified.** Performance was evaluated using Phase 1 only, and participants scoring $< 50\%$ of the Phase-1 maximum were excluded. Phase 1 directly assessed learning of the reward contingencies, consistent with the modelling assumption that reward contingencies were learned before the stimulation phase.

Additional Behavioural and Questionnaire Analysis

Distribution of Performance and Entropy

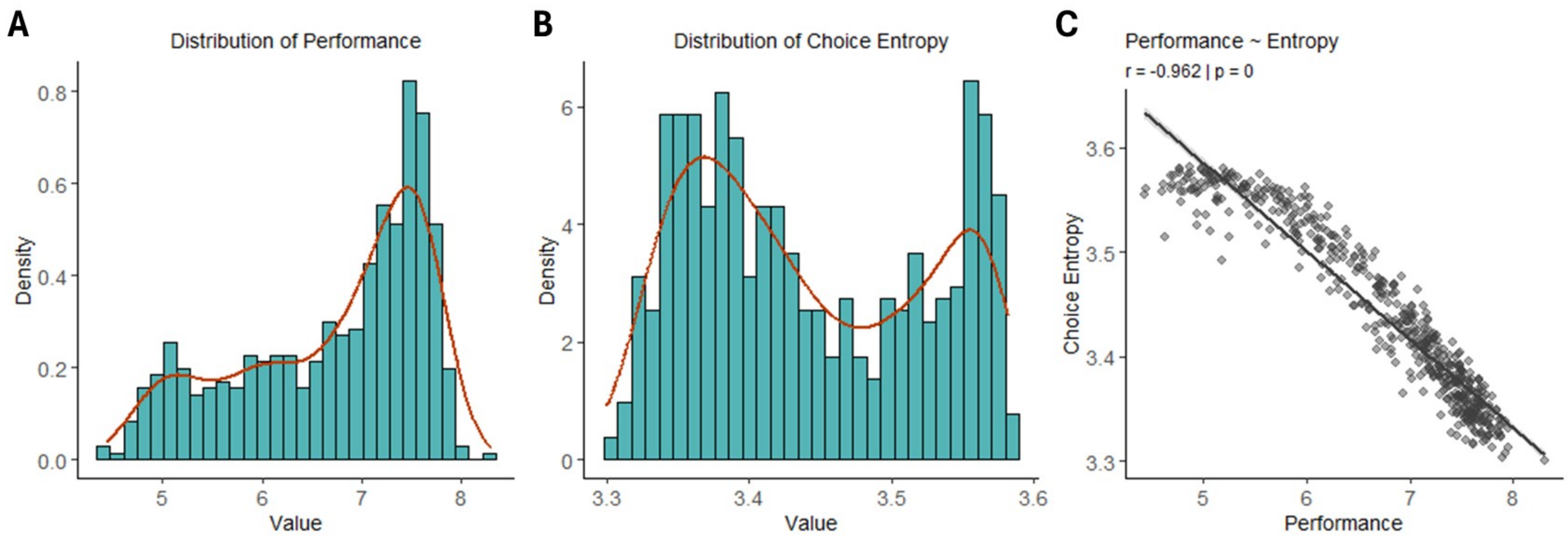

Figure S1. Distributions and association between performance and choice entropy. **(A)** Histogram and smoothed density of *Performance*. **(B)** Histogram and smoothed density of *Choice Entropy*. **(C)** Scatterplot of *Choice Entropy* versus *Performance* with linear fit (95% CI). Pearson correlation: $r = -0.962$, $p < 0.001$.

Effect of Traits on Round Performance

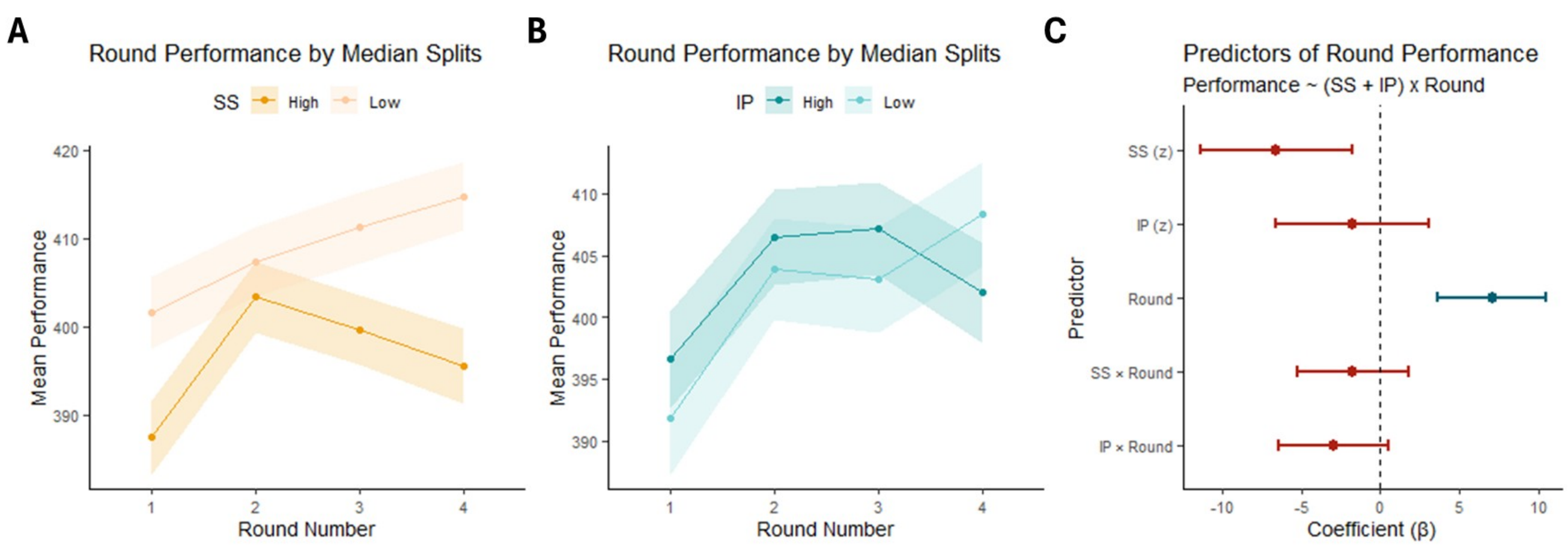

Figure S2. Round Performance × Trait. Mean total reward by round for median splits on **(A)** sensation-seeking (SS) and **(B)** impulsivity (IP); lines show group means, and shaded bands show $\pm SE$. **(C)** Fixed–effect coefficients (points) and 95% CIs (bars) from the mixed model $roundScore \sim (SS + IP) \times round + (1 \mid ID)$. We observed a negative association with SS ($\beta_{\mathrm{SS}} = -6.55$, $p = .008$) and a positive linear effect of round ($\beta_{\mathrm{round}} = 7.06$, $p < .001$), consistent with practice-related improvement, but no statistically significant main effect of IP ($\beta_{\mathrm{IP}} = -1.73$, $p = .48$). Neither the linear SS × round nor IP × round interaction reached statistical significance ($\beta_{\mathrm{SS}\times\mathrm{round(L)}} = -1.71$, $p = .34$; $\beta_{\mathrm{IP}\times\mathrm{round(L)}} = -2.94$, $p = .10$).

Effect of Traits on Reaction Times

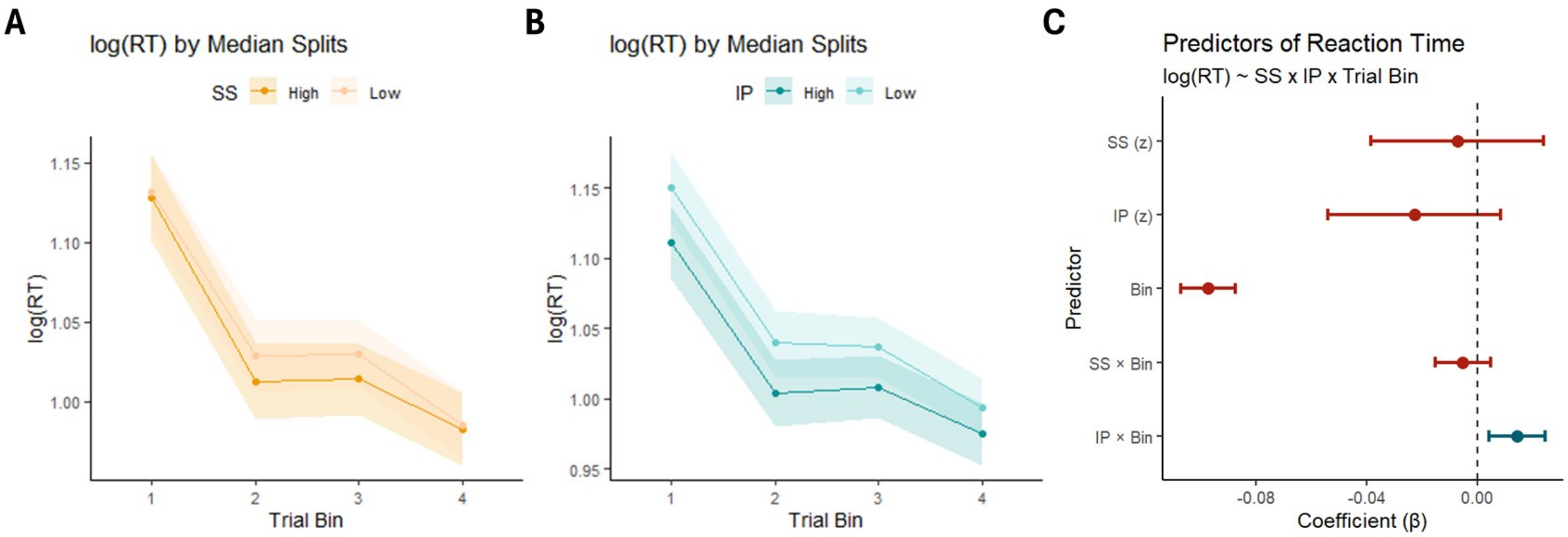

Figure S3. Reaction time by trait and trial bin.**(A)** Mean log(RT) by trial bin for median splits on sensation-seeking (SS); lines show group means, shaded bands show $\pm SE$.**(B)** Mean log(RT) by trial bin for median splits on Impulsivity (IP) with 95% CIs.**(C)** Fixed–effect coefficients (points) and 95% CIs (bars) from the mixed model $\log(\mathrm{RT}) \sim (\mathrm{SS}+\mathrm{IP}) \times \mathrm{Bin} + (1 \mid \mathrm{ID})$. RTs decreased across bins ($\beta_{bin} = -0.10$, $p < 0.001$), reflecting increasing efficiency, but there was no main effect of SS ($\beta_{SS} = -0.01$, $p = 0.65$) and no SS × bin interaction ($\beta_{SS\times bin} = -0.01$, $p = 0.31$). Thus, high-SS participants were generally neither slower nor faster, and they did not exhibit an atypical RT learning trajectory. IP scores showed no main effect on RTs ($\beta_{IP} = -0.02$, $p = 0.15$), although a significant IP × bin interaction ($\beta_{IP\times bin} = 0.01$, $p = 0.005$) indicated a modest modulation of RT dynamics without corresponding performance differences.

Effect of Traits on Performance with Omission Rate as Covariate

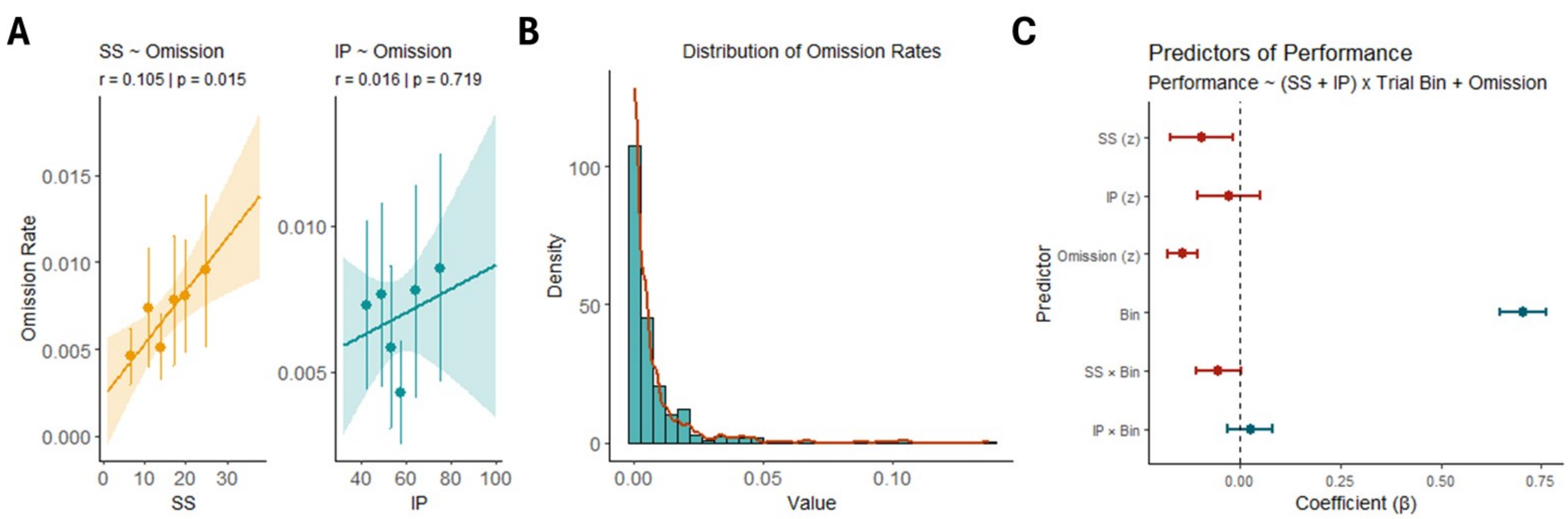

Figure S4. Omission rates and performance. **(A)** Relationship between omission rate and traits: sensation-seeking (SS) and Impulsivity (IP); points show participant means with 95% CIs and fitted linear trends. **(B)** Distribution of omission rates across trials/participants (histogram with smoothed density). **(C)** Fixed–effect coefficients (points) and 95% CIs (bars) from the mixed model $\mathrm{Performance} \sim (\mathrm{SS}+\mathrm{IP}) \times \mathrm{Bin} + \mathrm{Omission} + (1 \mid \mathrm{id})$, with standardised predictors. Omission rate robustly predicted poorer performance ($\beta_{missed} = -0.14$, $p < 0.001$), but SS crucially remained a significant negative predictor even after controlling for omissions ($\beta_{SS} = -0.10$, $p = 0.02$). Thus, increased misses contribute to, but do not fully explain, the SS-related performance cost.

Statistical Tables for Figures S2–S4

Predictor	Estimate	95% CI	Test statistic	p
**A. Round-wise performance**				
Sensation seeking	$\beta = -6.55$	$[-11.38, -1.73]$	$t(524) = -2.66$	.008
Impulsivity	$\beta = -1.73$	$[-6.56, 3.10]$	$t(524) = -0.70$	.483
Round (linear)	$\beta = 7.06$	$[3.61, 10.52]$	$t(1572) = 4.01$	$< .001$
Sensation seeking × Round (linear)	$\beta = -1.71$	$[-5.22, 1.79]$	$t(1572) = -0.96$	.338
Impulsivity × Round (linear)	$\beta = -2.94$	$[-6.44, 0.56]$	$t(1572) = -1.65$	.100
**B. Reaction time**				
Sensation seeking	$\beta = -0.007$	$[-0.038, 0.024]$	$t(524) = -0.45$	.650
Impulsivity	$\beta = -0.023$	$[-0.054, 0.008]$	$t(524) = -1.43$	.154
Trial bin (linear)	$\beta = -0.098$	$[-0.107, -0.088]$	$t(1572) = -19.21$	$< .001$
Sensation seeking × Trial bin (linear)	$\beta = -0.005$	$[-0.015, 0.005]$	$t(1572) = -1.01$	.311
Impulsivity × Trial bin (linear)	$\beta = 0.014$	$[0.004, 0.025]$	$t(1572) = 2.80$	.005
**C. Performance controlling for omission rate**				
Sensation seeking	$\beta = -0.097$	$[-0.175, -0.019]$	$t(516.1) = -2.44$	.015
Impulsivity	$\beta = -0.028$	$[-0.106, 0.050]$	$t(514.5) = -0.71$	.480
Omission rate	$\beta = -0.143$	$[-0.180, -0.105]$	$t(1890) = -7.48$	$< .001$
Trial bin (linear)	$\beta = 0.704$	$[0.647, 0.760]$	$t(1581) = 24.38$	$< .001$
Sensation seeking × Trial bin (linear)	$\beta = -0.054$	$[-0.110, 0.002]$	$t(1563) = -1.89$	.059
Impulsivity × Trial bin (linear)	$\beta = 0.025$	$[-0.031, 0.081]$	$t(1564) = 0.88$	.380
**D. Trait associations with omission rate**				
Sensation seeking	$\rho = 0.11$	$[0.02, 0.19]$	$S = 2.18 \times 10^7$	.015
Impulsivity	$\rho = 0.02$	$[-0.07, 0.10]$	$S = 2.40 \times 10^7$	.719

Table S2. Additional behavioural analyses. Panels A–C report fixed-effect estimates from linear mixed-effects models examining round-wise performance, reaction time, and performance after controlling for omission rate. Continuous trait predictors were standardised. Round and trial bin were entered as ordered factors; for concision, only the linear (L) polynomial contrasts are shown. Panel D reports Spearman rank correlations between trait scores and participant-level omission rates. Confidence intervals are 95% confidence intervals. p values for the mixed-effects models were obtained using Satterthwaite-approximated degrees of freedom. *Note.* $n = 527$ participants. Panels A–C were estimated using linear mixed-effects models with participant-specific random intercepts. Round and trial bin were entered as ordered factors using orthogonal polynomial contrasts; only the linear contrasts are displayed here for concision. Panel C additionally included standardised participant-level omission rate as a covariate. Panel D reports Spearman rank correlations; S denotes the Spearman test statistic. SS = sensation-seeking; IP = impulsivity.

Correlation Matrix of Questionnaires

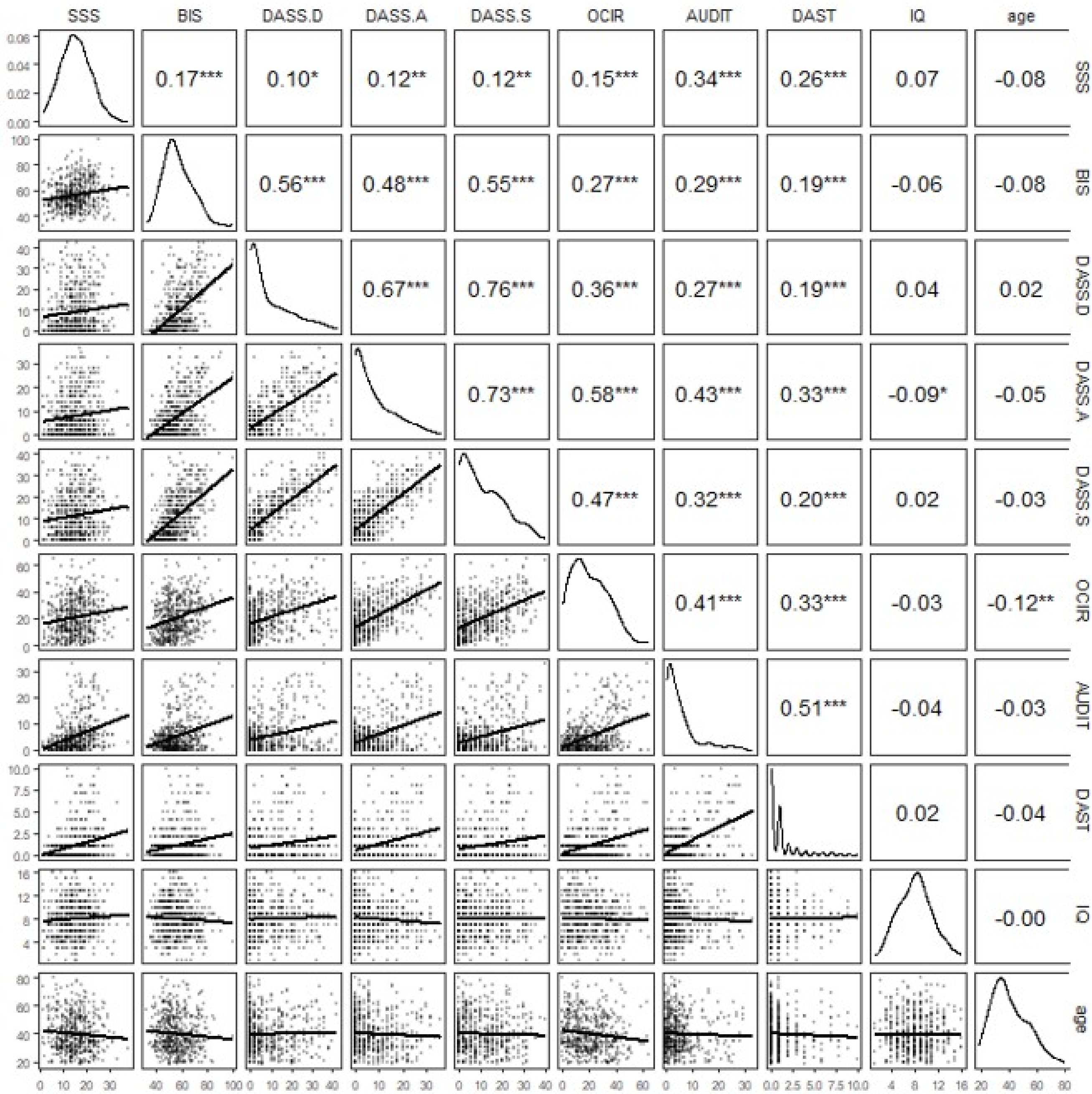

Figure S5. Correlation matrix for questionnaire measures. Upper triangle displays Pearson's r with significance stars ($^{***}p < .001$, $^{**}p < .01$, $^{*}p < .05$); diagonal panels show kernel density estimates; lower triangle shows scatterplot with a best fit line. Abbreviations: SSS = Sensation Seeking Scale; BIS = Barratt Impulsiveness Scale; DASS.D/A/S = Depression, Anxiety, and Stress subscales of the Depression Anxiety Stress Scales (DASS); OCIR = Obsessive-Compulsive Inventory-Revised (OCI-R); AUDIT = Alcohol Use Disorders Identification Test; DAST = Drug Abuse Screening Test; IQ = intelligence quotient.

Generalised Linear Mixed-Effects Modelling

Motor vs Stimulus Stickiness

We compared two forms of choice perseveration/ stickiness: a preregistered stimulus stickiness bonus based on the identity of the previous choice ($Sticky_s$, model 2) and a motor stickiness bonus based on the previous response side ($Sticky_m$, model 3). Motor stickiness provided a better overall fit, so we focus on $Sticky_m$ in subsequent analysis. We also evaluated models with random intercepts and random slopes for $R\mu$. Attempts to add random slopes for $S\mu$ and $S\sigma$ produced singular fits. Our best-fitting specification was Model 5, which we therefore used to test trait interactions in the next subsection.

model	AIC	BIC	RMSE	Formula
1	141153.9	141192.8	0.4369886	$choice \sim \Delta R\mu + \Delta S\mu + \Delta S\sigma$
2	141135.4	141184.1	0.4369726	$choice \sim \Delta R\mu + \Delta S\mu + \Delta S\sigma + Sticky_s$
3	141098.0	141146.7	0.4368896	$choice \sim \Delta R\mu + \Delta S\mu + \Delta S\sigma + Sticky_m$
4	139950.1	140008.6	0.4319289	$choice \sim \Delta R\mu + \Delta S\mu + \Delta S\sigma + Sticky_m + (1\|ID)$
5	131801.5	131879.4	0.4172918	$choice \sim \Delta R\mu + \Delta S\mu + \Delta S\sigma + Sticky_m + (1 + \Delta R\mu\|ID)$

Table S3. Model comparison for Experiment 2 predicting rightward choice (choice = 1 for "right"). All models include the core decision variables $\Delta R\mu$, $\Delta S\mu$, and $\Delta S\sigma$; optional history terms are Sticky_s (stimulus perseveration) and Sticky_m (motor perseveration). Random-effects structures are denoted $(1 \mid \text{ID})$ and $(1 + \Delta R\mu \mid \text{ID})$. Fit indices reported are AIC, BIC, and RMSE (all lower is better). Adding Sticky_m improves fit over Sticky_s (Model 3 vs. 2); allowing random intercepts improves fit (Model 4); the model with random intercepts and a random slope for $\Delta R\mu$ (Model 5) provides the best overall fit and is used for subsequent inference.

Interactions with Traits

We tested all candidate two- and three-way interactions between core decision variables and our traits of interest—sensation-seeking (SS) and impulsivity (IP). Significant interactions are reported in the main text; complete model outputs (all coefficients β weights) are provided in Fig. S7. To assess model specification, we used DHARMa simulation-based residual diagnostics in R, including checks for dispersion, outliers, and normality (Fig. S6).

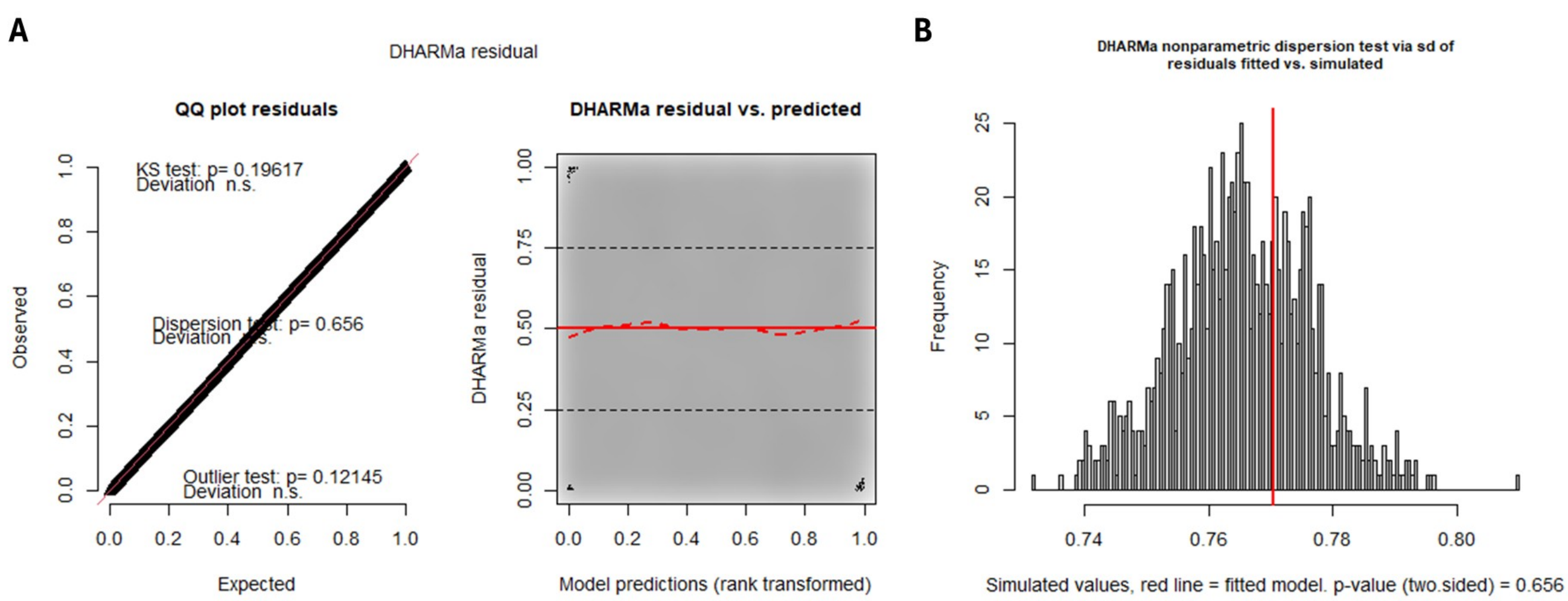

Figure S6. DHARMa diagnostics for the Predictors × Trait GLMM (1,000 simulations). (A) Left: QQ plot of scaled residuals indicates approximate uniformity (KS test, $p = 0.20$). Right: residuals vs. fitted values with DHARMa quantile curves show no systematic pattern; dispersion test n.s. ($p = 0.66$). (B) Nonparametric dispersion check: distribution of simulated residual SDs with the fitted-model value (red line); $p = 0.66$. Outlier test n.s. ($p = 0.12$)

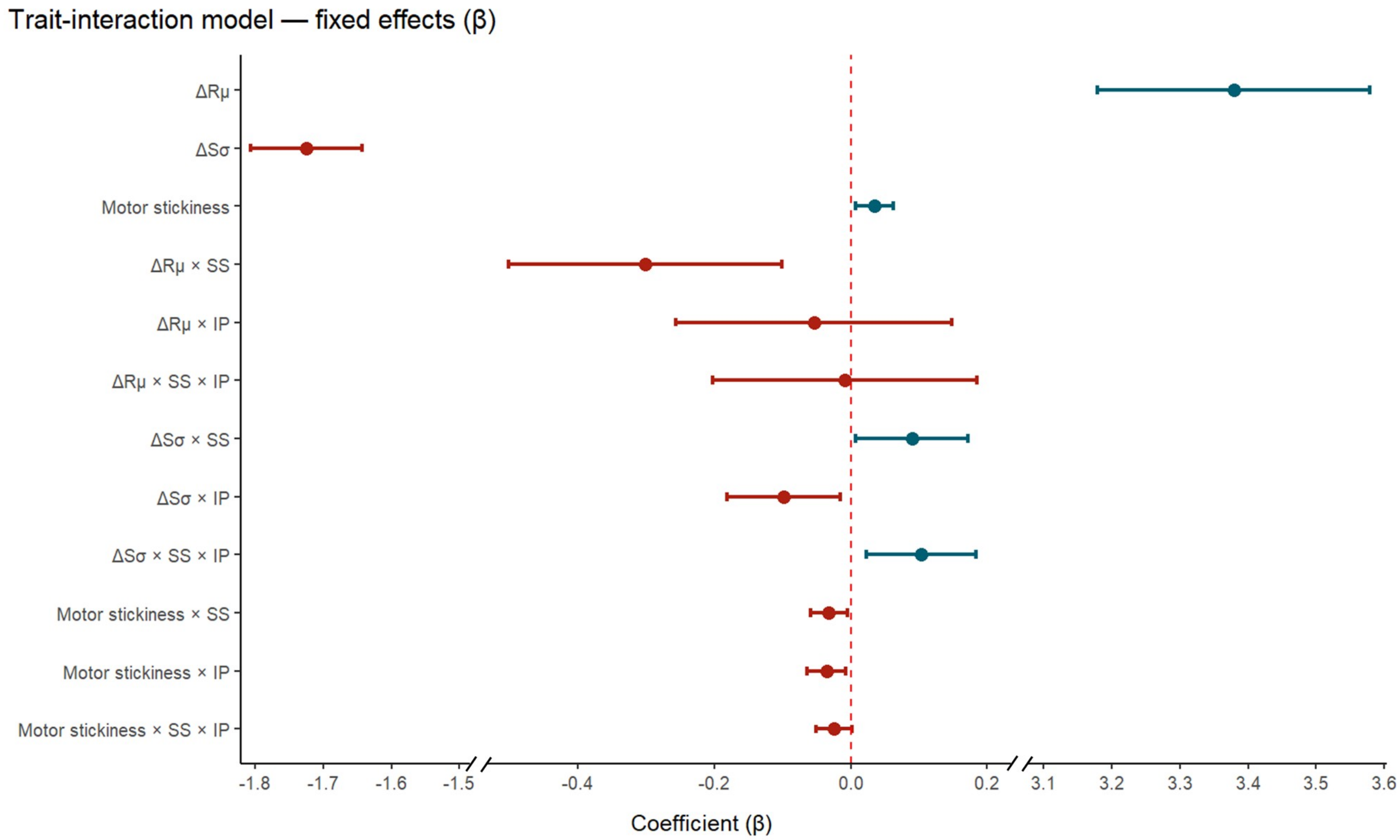

Figure S7. Fixed-effect coefficients (β) from the Trait–Interaction GLMM (binomial link). Points show estimates and horizontal bars show 95% CIs for the main effects of the decision variables ($\Delta R\mu$, $\Delta S\sigma$, Motor stickiness) and their interactions with traits—sensation-seeking (SS) and impulsivity (IP)—including two-way and three-way terms (e.g., $\Delta R\mu \times SS$, $\Delta R\mu \times IP$, $\Delta R\mu \times SS \times IP$). The vertical dashed line marks $\beta = 0$; effects whose CIs do not cross zero are considered statistically reliable. Random effects: intercepts and by-participant slopes for $\Delta R\mu$.

Description of Reduced Models

We outline here the reduced models introduced in the main text. These models differ only in their choice rule. For each bandit j at time t, the Q-value $Q_{j,t}$ was defined as a linear combination of the estimated underlying point payoff $R\mu_{j,t}$ and, depending on the model, the stimulation-related terms: the estimated mean probability of stimulation $S\mu_{j,t}$, weighted by free parameter θ, and the associated uncertainty $S\sigma_{j,t}$, weighted by free parameter ω. All Q-values were scaled by the temperature parameter τ:

R-only

$$Q_{j,t} = \frac{R\mu_{j,t}}{\tau} \tag{S1}$$

R-θ

$$Q_{j,t} = \frac{R\mu_{j,t} + \theta S\mu_{j,t}}{\tau} \tag{S2}$$

R-ω

$$Q_{j,t} = \frac{R\mu_{j,t} + \omega S\sigma_{j,t}}{\tau} \tag{S3}$$

R-$\theta\omega$

$$Q_{j,t} = \frac{R\mu_{j,t} + \theta S\mu_{j,t} + \omega S\sigma_{j,t}}{\tau} \tag{S4}$$

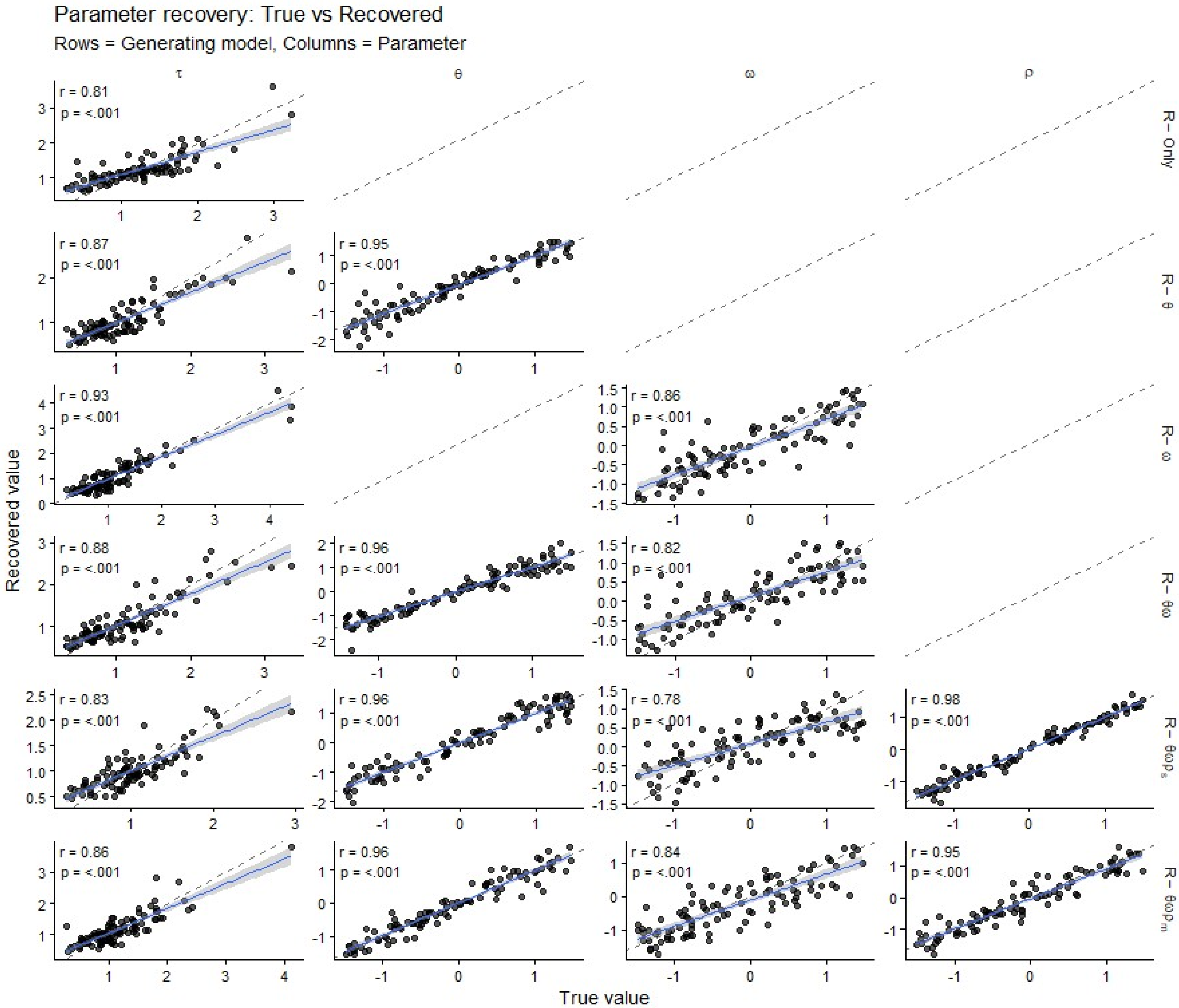

Figure S8. Scatter plots compare true (x-axis) vs recovered posterior mean (y-axis) parameters for a synthetic dataset ($N = 100$). Rows are generating models (R–Only, R–θ, R–ω, R–$\theta\omega$, R–$\theta\omega\rho_s$, R–$\theta\omega\rho_m$); columns are parameters (τ, θ, ω, ρ). The dashed line shows the identity (y = x); the solid line is a regression line fit with 95% CI. Panel insets report Pearson's r and corresponding p-values. Blank panels indicate parameters not present in that generating model.

Model	Parameter	Mean	95% CrI	ESS	$\widehat{R}$
**Experiment 1**					
$R-\theta$	θ	-0.1003	$[-0.1725, -0.0270]$	933.1	1.0031
	$1/\tau$ (inverse temperature)	5.7552	[4.9597, 6.6187]	3443.2	1.0009
Norbury (2015)	θ	-0.0545	$[-0.0991, -0.0090]$	3865.5	1.0009
	$1/\tau$ (inverse temperature)	5.9838	[5.1766, 6.8483]	3967.9	1.0011
$R-\omega$	ω	-2.2303	$[-2.5846, -1.8873]$	3865.5	1.0009
	$1/\tau$ (inverse temperature)	6.3080	[5.5327, 7.1192]	3967.9	1.0011
**Experiment 2**					
R-only	$1/\tau$ (inverse temperature)	3.5384	[3.2061, 3.9031]	562.4	1.0061
$R-\theta$	θ	0.0039	[0.0007, 0.0072]	8663.4	1.0002
	$1/\tau$ (inverse temperature)	3.5229	[3.1805, 3.8906]	488.4	1.0061
$R-\omega$	ω	-0.2254	$[-0.2468, -0.2049]$	3269.4	1.0001
	$1/\tau$ (inverse temperature)	3.2062	[2.9368, 3.4887]	466.5	1.0057
$R-\theta\omega$	θ	0.0050	[0.0009, 0.0091]	8893.2	1.0005
	ω	-0.2253	$[-0.2463, -0.2050]$	4428.4	1.0002
	$1/\tau$ (inverse temperature)	3.2233	[2.9567, 3.5079]	623.4	1.0045
$R-\theta\omega\rho_s$	θ	0.0050	[0.0009, 0.0091]	10113.0	0.9998
	ω	-0.2287	$[-0.2504, -0.2075]$	6259.8	1.0002
	$1/\tau$ (inverse temperature)	3.2364	[2.9505, 3.5311]	889.8	1.0034
	ρ	-0.0398	$[-0.0807, 0.0009]$	11754.7	0.9998
$R-\theta\omega\rho_m$	θ	0.0051	[0.0010, 0.0092]	9325.2	0.9997
	ω	-0.2267	$[-0.2482, -0.2066]$	5344.1	1.0001
	$1/\tau$ (inverse temperature)	3.3020	[3.0366, 3.5893]	781.4	1.0041
	ρ	0.0421	[0.0022, 0.0827]	3064.2	1.0001

Table S4. Group-level posterior parameter estimates across computational models. Values are posterior means and 95% credible intervals (CrIs) for group-level parameter estimates. Temperature as reported in the main text was obtained by taking the reciprocal of the inverse-temperature parameter estimated by the model reported here, such that larger values indicate greater choice stochasticity. ESS = effective sample size; ρ_s = stimulus stickiness; ρ_m = motor stickiness.

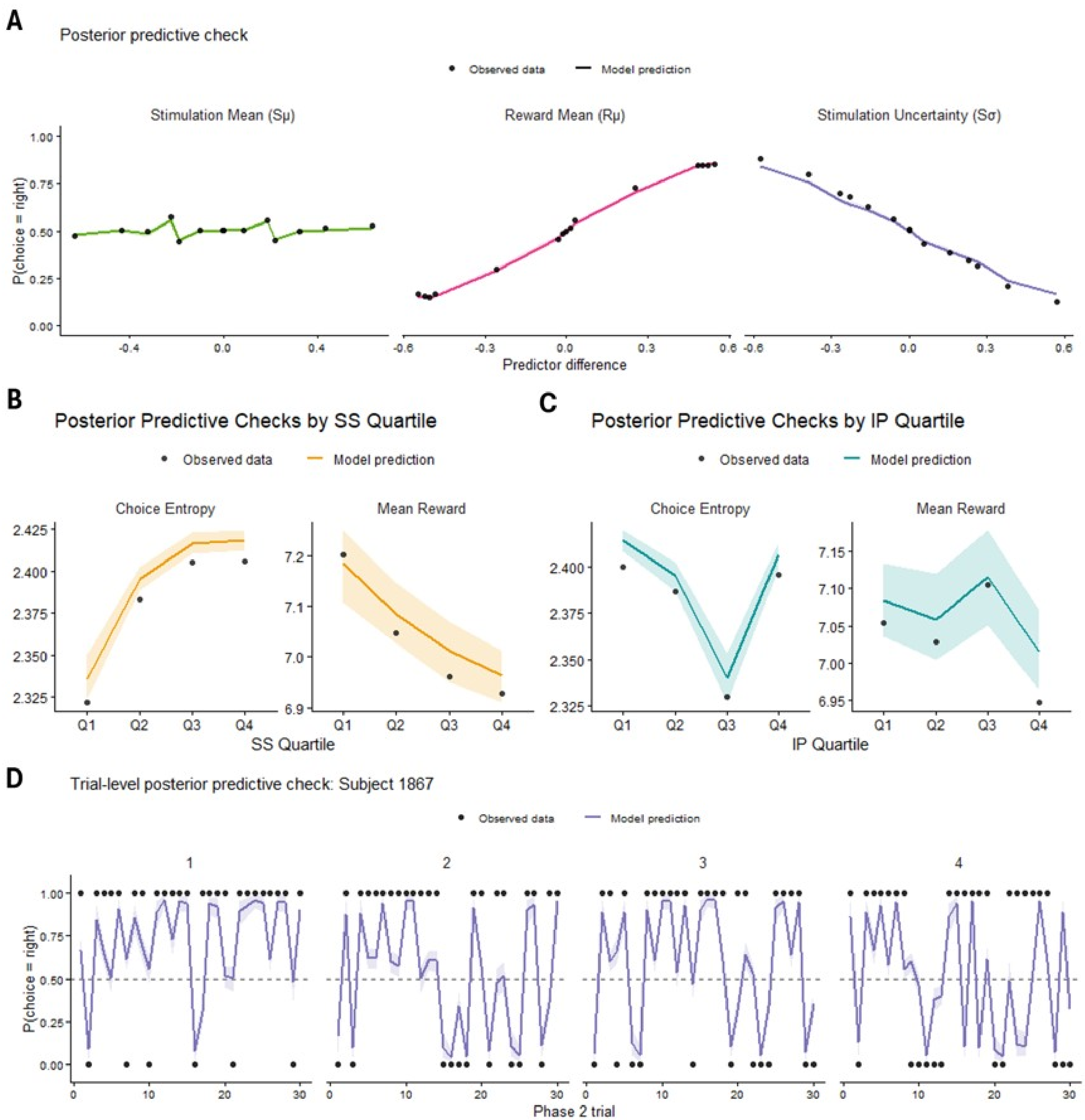

Figure S9. Posterior predictive checks for the winning hierarchical model (R–$\theta\omega\rho_m$). (A) Observed and model-predicted choice behaviour across the three main decision variables: stimulation mean (Sμ), reward mean (Rμ), and stimulation uncertainty (Sσ). Points show observed choice proportions after binning trials by predictor difference; coloured lines show posterior predictive means. (B–C) Posterior predictive checks for subject-level behavioural summaries across sensation-seeking (SS; B) and impulsivity (IP; C) quartiles. Points show observed group means, lines show posterior predictive means, and shaded ribbons show 95% posterior predictive intervals. (D) Example trial-level posterior predictive check for one random participant. The line shows the posterior mean predicted probability of choosing the right option on each Phase 2 trial; the shaded band shows the 95% posterior interval; and the black points show observed choices coded as left (0) and right (1). Together, these checks show that the model captured both trial-level choice sensitivity to reward, expected stimulation, and stimulation uncertainty, as well as key trait-binned behavioural summaries.

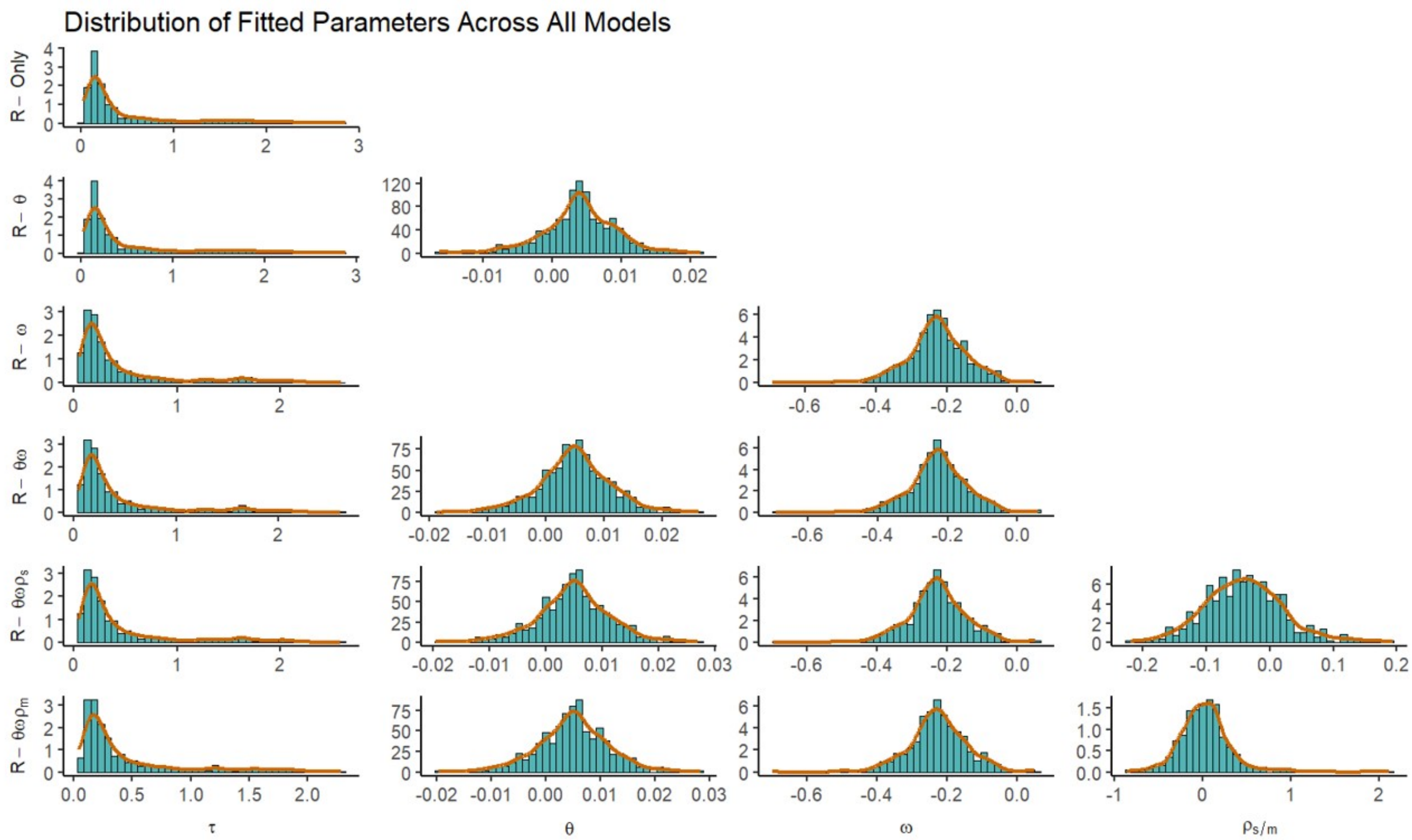

Figure S10. Distributions of fitted parameters across computational models. Columns show participant-level parameter estimates for expected-stimulation weighting (θ), stimulation-uncertainty weighting (ω), stickiness (ρ), and choice temperature (τ); rows correspond to model variants. Histograms show the distribution of participant-level posterior estimates, with overlaid kernel density curves. For models including stimulus stickiness, ρ_s denotes the bonus for repeating the previously chosen stimulus; for models including motor stickiness, ρ_m denotes the bonus for repeating the previous response side.

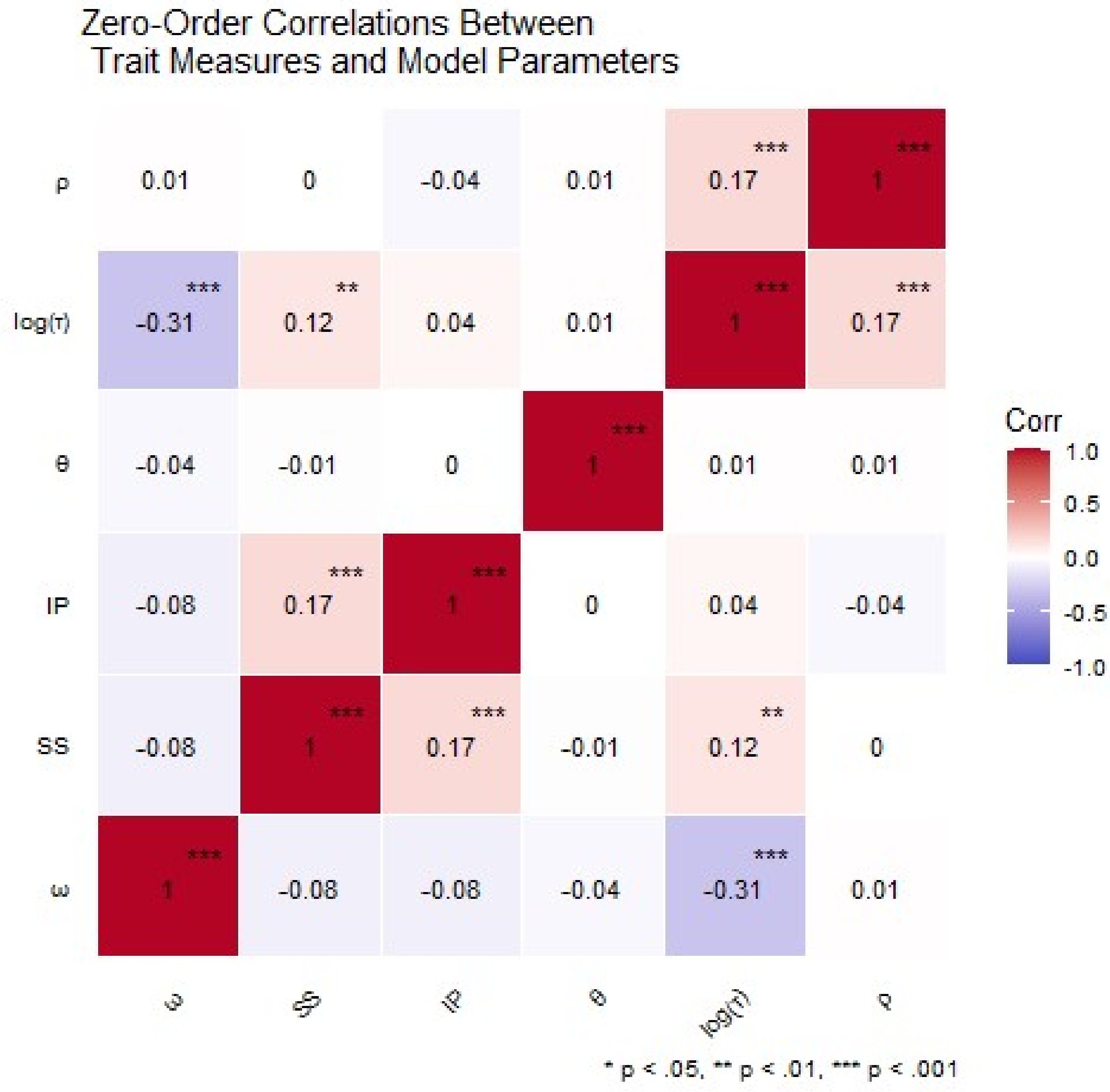

Figure S11. Zero-order correlations between sensation-seeking (SS), impulsivity (IP), and computational model parameters. Values indicate Pearson correlation coefficients (r). Asterisks denote statistically significant associations ($^{*}p < .05$, $^{**}p < .01$, $^{***}p < .001$). Model parameters include expected stimulation (θ), uncertainty (ω), choice stochasticity ($\log(\tau)$), and motor stickiness (ρ).

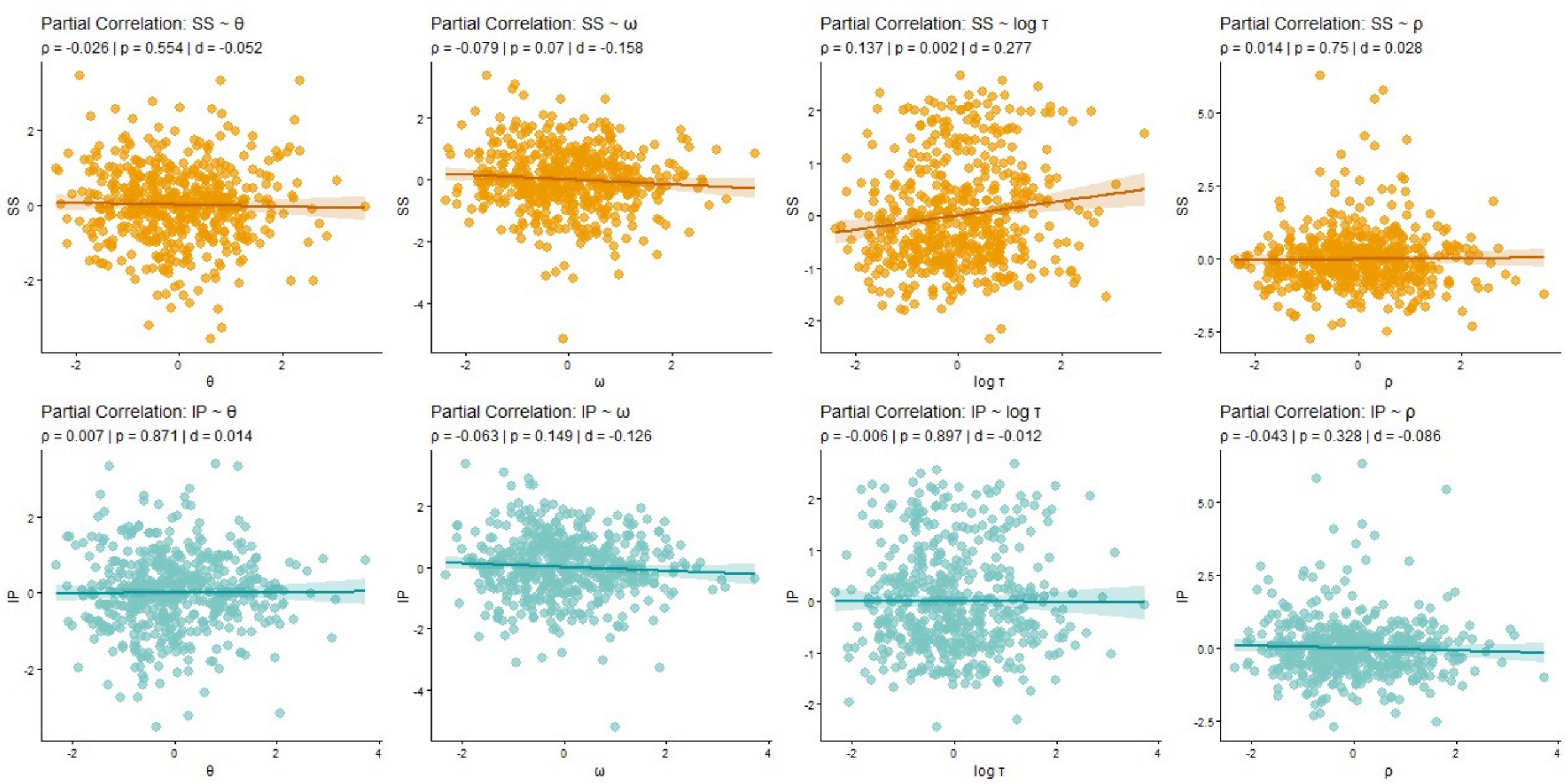

Figure S12. Partial correlations between trait measures and computational model parameters. The top row shows associations with sensation-seeking (SS), controlling for impulsivity (IP), age, IQ, and gender; the bottom row shows associations with impulsivity, controlling for sensation-seeking, age, IQ, and gender. Columns show associations with expected stimulation (θ), uncertainty (ω), choice stochasticity ($\log(\tau)$), and motor stickiness (ρ), respectively. Points represent residualised individual-level values after accounting for the corresponding covariates, with lines showing linear fits and shaded areas indicating 95% confidence intervals.

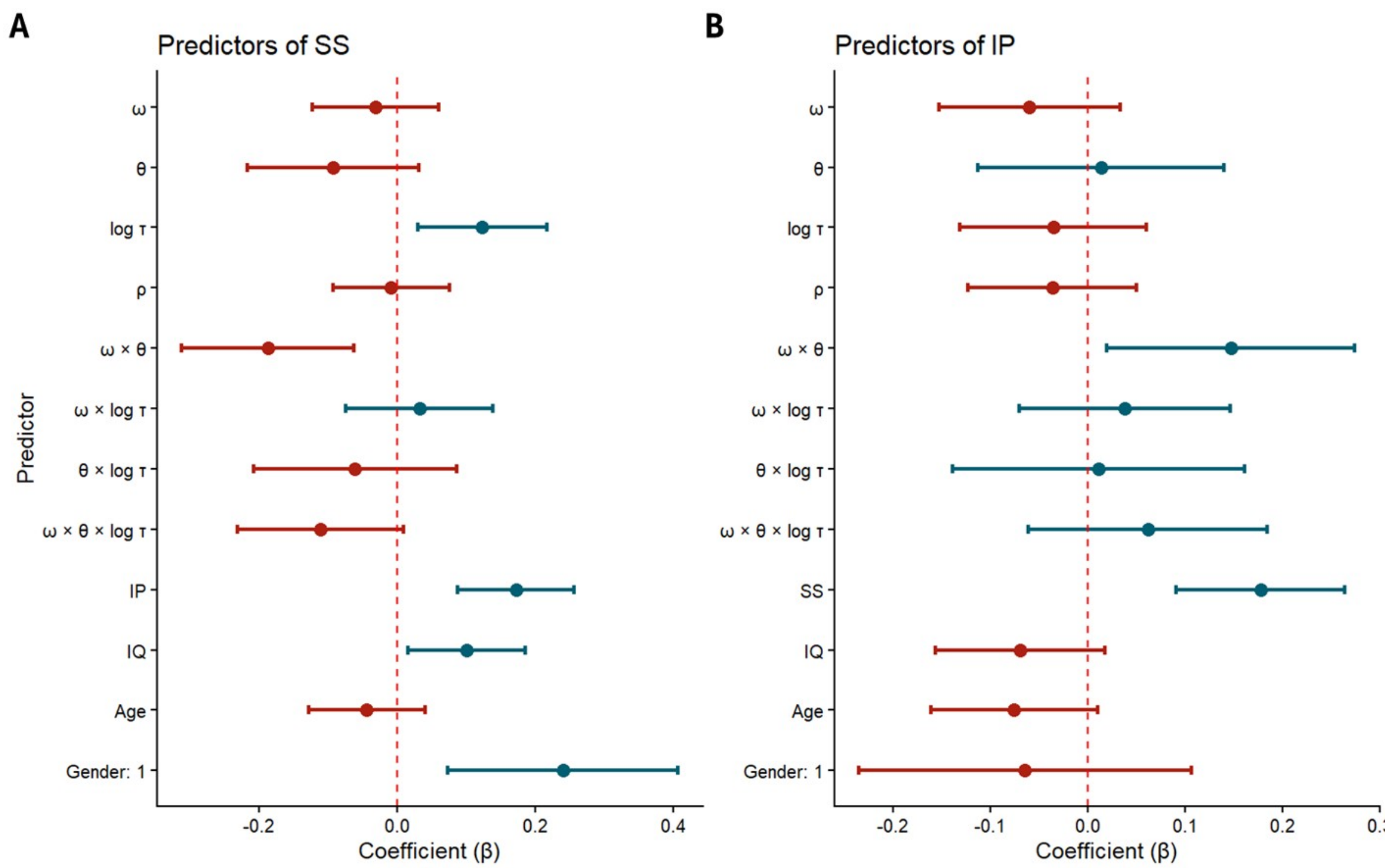

Figure S13. Regression of traits on computational parameters and covariates. Points show fixed–effect coefficients (β) with 95% CIs; the vertical dashed line marks $\beta = 0$. **(A)** Predictors of sensation-seeking (SS). **(B)** Predictors of Impulsivity (IP). Predictors include model parameters ω, θ, and $\log\tau$, the stickiness parameter ρ (additive main effect), and covariates (IP or SS, IQ, Age, Gender). Interaction terms were limited to $\omega \times \theta$, $\omega \times \log\tau$, $\theta \times \log\tau$, and $\omega \times \theta \times \log\tau$. *Note:* We did not include ρ in the interaction terms, as this was not theoretically motivated and we sought to avoid unnecessary expansion of the interaction space.

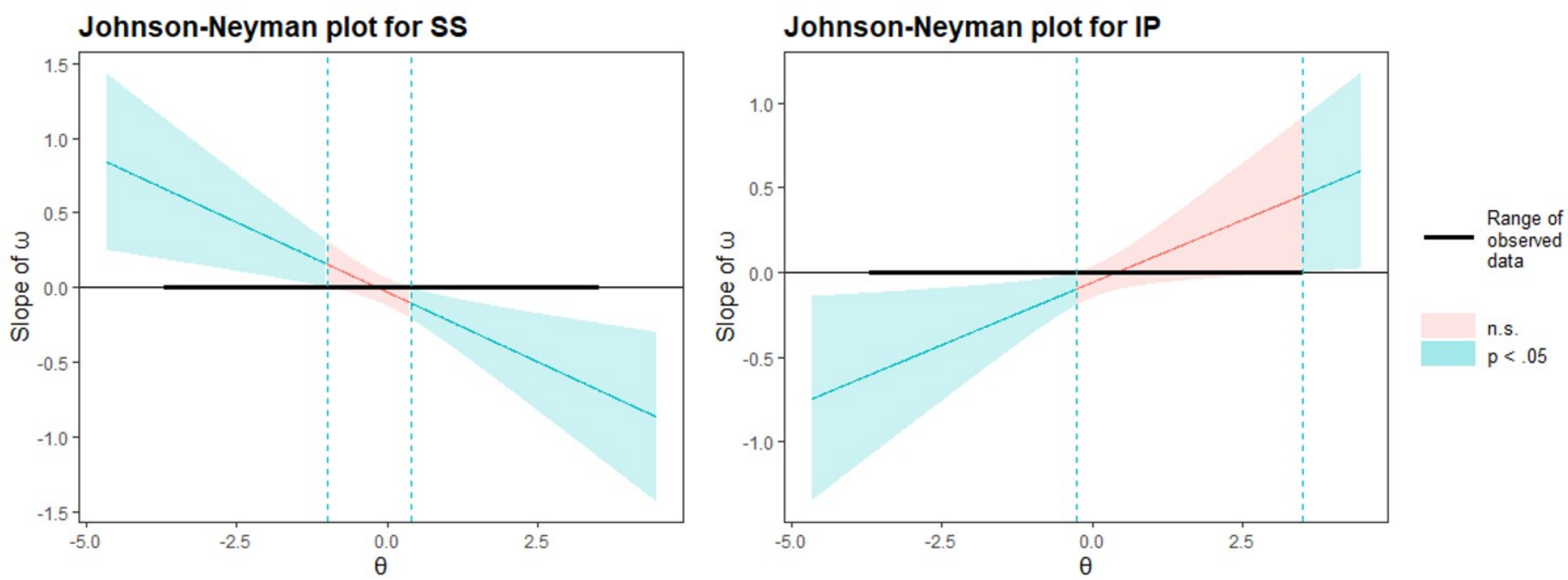

Figure S14. Johnson–Neyman analysis of the ($\theta \times \omega$) interaction predicting sensation-seeking (SS) and impulsivity (IP). The x-axis shows expected-stimulation weighting (θ), and the y-axis shows the conditional slope of uncertainty weighting (ω) on each trait. Solid lines show the estimated conditional effect of ω across values of θ, with shaded bands indicating the 95% confidence interval. Cyan regions indicate values of θ where the conditional slope of ω is statistically significant ($p < .05$); pink regions indicate non-significant regions. Vertical dashed lines mark the Johnson–Neyman bounds, and the black horizontal bar indicates the observed range of θ in the data. For SS, ω was positively associated with SS at lower values of θ, but negatively associated with SS at higher values of θ. For IP, a higher ω was associated with lower IP primarily when θ was low, suggesting a distinct parameter-interaction profile across the two traits.

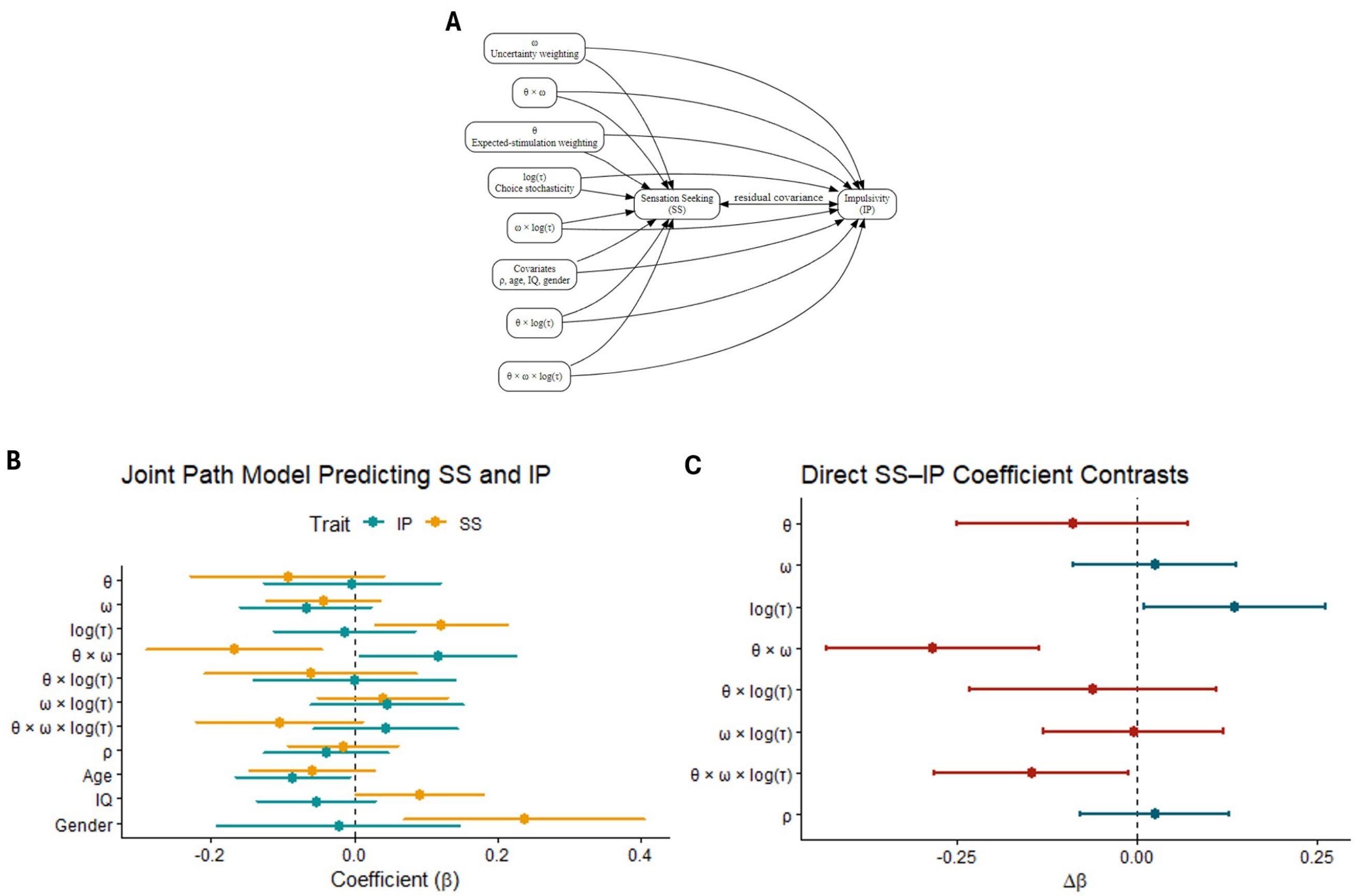

Figure S15. Joint path model comparing parameter associations with sensation-seeking (SS) and impulsivity (IP). (A) Schematic of the saturated joint path model. SS and IP were simultaneously regressed on the same computational parameters and interaction terms: uncertainty weighting (ω), expected-stimulation weighting (θ), choice stochasticity ($\log \tau$), their two-way and three-way interactions, and covariates including stickiness (ρ), age, IQ, and gender. The residual covariance between SS and IP was freely estimated. (B) Path coefficients predicting SS and IP. Points show regression coefficients (β), and horizontal bars show 95% confidence intervals. (C) Direct SS–IP coefficient contrasts, computed as the SS coefficient minus the IP coefficient for each corresponding path. Points show contrast estimates ($\Delta\beta$), and horizontal bars show 95% confidence intervals. Negative values indicate that the corresponding association was lower for SS than for IP. Because the joint path model was saturated, global fit indices were not interpreted; inference focused on coefficient estimates and direct SS–IP contrasts.

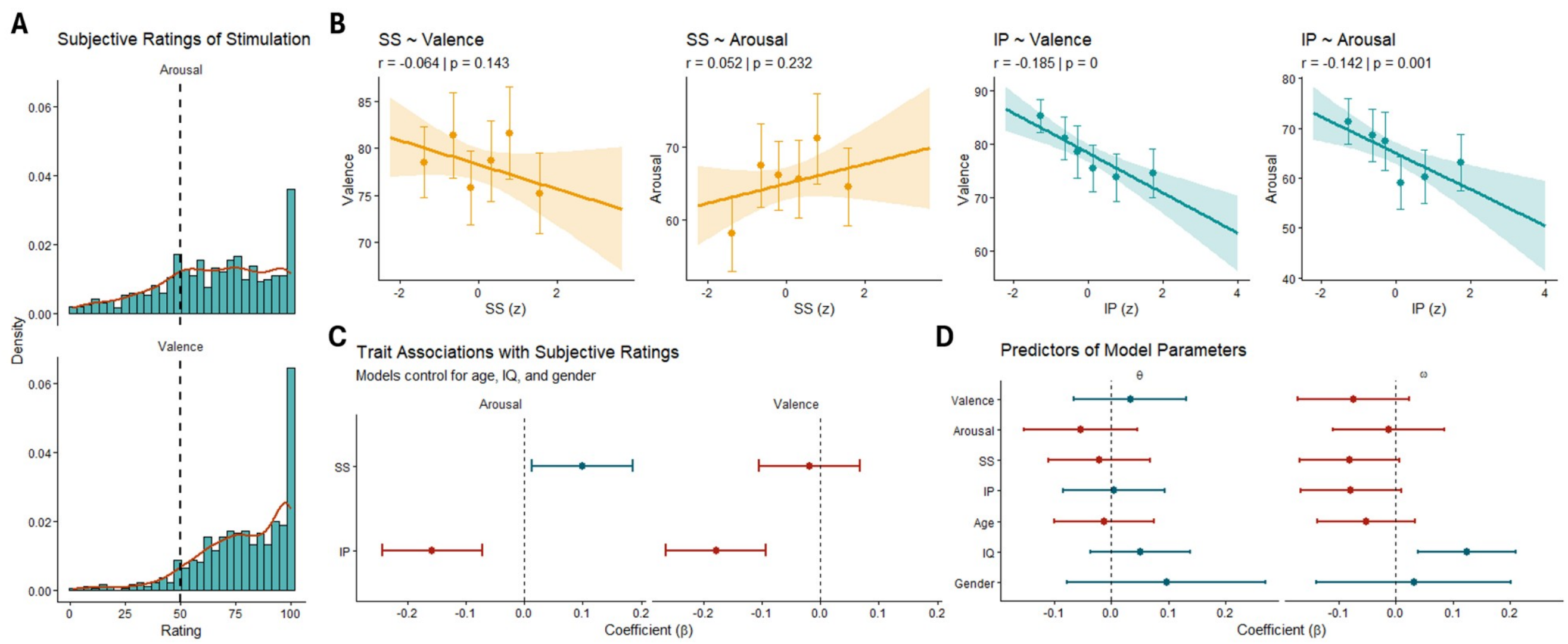

Figure S16. Subjective valence and arousal ratings of the stimulation. (A). Distributions of participants' mean arousal and valence ratings. Dashed vertical lines indicate the scale midpoint. Participants generally rated the stimulation as pleasant and moderately arousing. (B). Binned associations between sensation-seeking (SS) and impulsivity (IP) with subjective valence and arousal ratings. Points show binned means with error bars, solid lines show fitted linear trends, and shaded regions show 95% confidence intervals. SS was not significantly associated with valence or arousal in the binned correlations, whereas IP was negatively associated with both ratings. (C). Standardised regression coefficients predicting subjective arousal and valence from SS and IP, controlling for age, IQ, and gender. Error bars show 95% confidence intervals. (D). Standardised regression coefficients predicting expected-stimulation weighting (θ) and uncertainty weighting (ω) from valence, arousal, traits, and covariates. Valence and arousal did not reliably predict either computational parameter, suggesting that the parameter-level trait effects were not explained by subjective ratings of the stimulation.
