# Supplementary material for: Stimulation uncertainty reveals distinct mechanisms of sensation seeking and impulsivity
Source: Commun Psychol. 2026 Oct 23;4:141. doi: 10.1038/s44271-026-00525-5 (PMC13694268; doi:10.1038/s44271-026-00525-5)
Supplement: Supplementary file 2 [file 44271_2026_525_MOESM2_ESM.pdf]

nature portfolio

Peer Review File

Stimulation Uncertainty Reveals Distinct Mechanisms of Sensation Seeking and Impulsivity

Corresponding Author: Dr Ern Wong

This file contains all reviewer reports in order by version, followed by all author rebuttals in order by version.

Version 0:

Reviewer comments:

Reviewer #1

(Remarks to the Author)
I have previously reviewed this manuscript at Nature Communications, and the authors have provided a direct and detailed response letter. I am delighted to see that all my concerns have been addressed. In my view, the manuscript is strongly improved, easier to read and understand, and will make an important contribution to the literature.

Reviewer #2

(Remarks to the Author)
The authors have adequately responded to my comments.

Open Access This Peer Review File is licensed under a Creative Commons Attribution 4.0 International License, which permits use, sharing, adaptation, distribution and reproduction in any medium or format, as long as you give appropriate credit to the original author(s) and the source, provide a link to the Creative Commons license, and indicate if changes were made.
In cases where reviewers are anonymous, credit should be given to 'Anonymous Referee' and the source.

The images or other third party material in this Peer Review File are included in the article's Creative Commons license, unless indicated otherwise in a credit line to the material. If material is not included in the article's Creative Commons license and your intended use is not permitted by statutory regulation or exceeds the permitted use, you will need to obtain permission directly from the copyright holder.
To view a copy of this license, visit https://creativecommons.org/licenses/by/4.0/

Response to Reviewers

Dear Editor,

We sincerely thank you and the reviewers for your careful consideration of our revised manuscript, *"Stimulation Uncertainty Reveals Distinct Mechanisms of Sensation Seeking and Impulsivity."*

We are grateful that both reviewers found that their previous concerns had been adequately addressed. We especially appreciate Reviewer #1's positive assessment that the manuscript has been substantially improved and that it makes an important contribution to the literature.

We again thank the Editor and Reviewers for their time, thoughtful feedback, and constructive guidance throughout the review process.

Sincerely,

Ern Wong, Tobias U. Hauser, Aswath Chandrasekaran, Pietro Pietrini, & Charley M. Wu
